# Economic evaluation of type 2 diabetes prevention programmes: Markov model of low- and high-intensity lifestyle programmes and metformin in participants with different categories of intermediate hyperglycaemia

**DOI:** 10.1186/s12916-017-0984-4

**Published:** 2018-01-30

**Authors:** Samantha Roberts, Dawn Craig, Amanda Adler, Klim McPherson, Trisha Greenhalgh

**Affiliations:** 1 Nuffield Department of Primary Care Health Sciences, University of Oxford, Radcliffe Primary Care Building, Radcliffe Observatory Quarter, Woodstock Road, Oxford, OX2 6GG UK; 2 Institute of Health & Society, University of Newcastle, Richardson Road, Newcastle Upon Tyne, NE1 7RU UK; 3 Addenbrooke’s Hospital, Hills Road, Cambridge, CB2 0QQ UK

**Keywords:** Diabetes prevention, Prediabetes, Intermediate hyperglycaemia, Economic evaluation, Impaired fasting glucose, Impaired glucose tolerance, HbA1c in at-risk range, Cost-effective

## Abstract

**Background:**

National guidance on preventing type 2 diabetes mellitus (T2DM) in the UK recommends low-intensity lifestyle interventions for individuals with intermediate categories of hyperglycaemia defined in terms of impaired fasting glucose (IFG) or ‘at-risk’ levels of HbA1c. In a recent systematic review of economic evaluations of such interventions, most studies had evaluated intensive trial-based lifestyle programmes in participants with impaired glucose tolerance (IGT). This study examines the costs and effects of different intensity lifestyle programmes and metformin in participants with different categories of intermediate hyperglycaemia.

**Methods:**

We developed a decision tree and Markov model (50-year horizon) to compare four approaches, namely (1) a low-intensity lifestyle programme based on current NICE guidance, (2) a high-intensity lifestyle programme based on the US Diabetes Prevention Program, (3) metformin, and (4) no intervention, modelled for three different types of intermediate hyperglycaemia (IFG, IGT and HbA1c). A health system perspective was adopted and incremental analysis undertaken at an individual and population-wide level, taking England as a case study.

**Results:**

Low-intensity lifestyle programmes were the most cost-effective (£44/QALY, £195/QALY and £186/QALY compared to no intervention in IGT, IFG and HbA1c, respectively). Intensive lifestyle interventions were also cost-effective compared to no intervention (£2775/QALY, £6820/QALY and £7376/QALY, respectively, in IGT, IFG and HbA1c). Metformin was cost-effective relative to no intervention (£5224/QALY, £6842/QALY and £372/QALY in IGT, IFG and HbA1c, respectively), but was only cost-effective relative to other treatments in participants identified with HbA1c. At a willingness-to-pay threshold of £20,000/QALY, low- and high-intensity lifestyle programmes were cost-effective 98%, 99% and 98% and 81%, 81% and 71% of the time in IGT, IFG and HbA1c, respectively. An England-wide programme for 50–59 year olds could reduce T2DM incidence by < 3.5% over 50 years and would cost 0.2–5.2% of the current diabetes budget for 2–9 years.

**Discussion:**

This analysis suggests that current English national policy of low-intensity lifestyle programmes in participants with IFG or HbA1c will be cost-effective and have the most favourable budget impact, but will prevent only a fraction of cases of T2DM. Additional approaches to prevention need to be investigated urgently.

**Electronic supplementary material:**

The online version of this article (doi:10.1186/s12916-017-0984-4) contains supplementary material, which is available to authorized users.

## Background

Diabetes mellitus is a global health priority, with a high prevalence (9% of adults globally are estimated to have the disease) and a substantial economic burden (accounting for 12% of global health expenditure). Cost is predicted to rise from $1.197 billion in 2015 to $1.452 billion by 2040 due to the increased prevalence of risk factors for diabetes, such as obesity, and the ageing of the world’s population [1]. By 2040, according to current trends, prevalence could be 642 million [1].

A number of large trials in the US [2], China [3], Finland [4] and India [5] have shown that type 2 diabetes mellitus (T2DM) can be prevented or delayed through lifestyle programmes or metformin in individuals with measures of glycaemia lower than those required to diagnose diabetes, but higher than ‘normal’. Lifestyle programmes included in these trials were intensive and sustained, provided by specialist staff over 3–10 years. Subsequent translation of these programmes into ‘real-world’ settings led to shorter programmes (3–24 months long) delivered by non-specialist staff, with more limited impact on the incidence of T2DM [6, 7].

Participants for diabetes prevention programmes are identified by the presence of ‘prediabetes’ or intermediate hyperglycaemia (measures of glycaemia lower than those required to diagnose T2DM, but higher than ‘normal’) or an assessment of risk of developing diabetes in the future (e.g. through the use of diabetes risk scores) [8]. Intermediate hyperglycaemia is a generic term that includes impaired fasting glucose (IFG), impaired glucose tolerance (IGT) and HbA1c in the ‘at-risk’ range. These different types of prediabetes differ in terms of their physiology, prevalence, progression to T2DM and their response to prevention programmes [9–12]. For example, while the evidence base for diabetes prevention among people with IGT is robust, few interventional studies exist for participants with isolated IFG and, to our knowledge, no randomised controlled trials have examined the effect on progression to T2DM in participants with isolated HbA1c in the at-risk range.

Given the increasing impact on populations and health budgets, the burden of T2DM is a key issue for policymakers. Diabetes prevention guidance issued by the National Institute of Clinical Excellence (NICE) in the UK and the Preventative Services Task Force in the US favours low-intensity lifestyle programmes [13, 14], focused on participants with IFG or ‘at-risk’ HbA1c in the UK. However, our recent systematic review [15] showed that there are few economic evaluations of these type of interventions, and the majority of those that do exist use treatment effects drawn from the trials evaluating more intensive lifestyle programmes in participants with IGT. To date, the generalisability of this assumption has not been validated. Additionally, no evaluation, to our knowledge, compares a pragmatic lifestyle programme with metformin or programmes for participants with ‘at-risk’ HbA1c with those offered to participants with other types of intermediate hyperglycaemia.

### Research question

This study evaluates the gap between existing evidence and current policy, exploring (1) the impact of the type of prediabetes chosen as the entry criteria for a programme, (2) the role of metformin versus low-intensity lifestyle programmes, and (3) the impact of the intensity of the lifestyle programme offered. This was analysed by modelling the cost and consequences (in terms of quality-adjusted life-years (QALY), incident cases of T2DM and average number of years with T2DM) for:

1. Three different definitions of intermediate hyperglycaemia (IFG, HbA1c, IGT) used to select participants for diabetes prevention programmes, and
2. Three types of diabetes prevention programme (metformin, intensive trial-based lifestyle programme, low-intensity pragmatic lifestyle programme)

A number of economic evaluations of lifestyle programmes and metformin for the prevention of diabetes have been undertaken [16–19]. To our knowledge, this is the first to compare (1) differences between participants with IFG, IGT and HbA1c, and (2) different intensities of lifestyle intervention with metformin. In addition, this is the first review to utilise data from recent meta-analyses of treatment effects in randomised controlled trials for lifestyle programmes [8, 15, 20, 21].

## Methods

A de novo economic model (decision tree and Markov model) was developed in TreeAgePro (TreeAge Software Inc.). An NHS perspective was adopted for the analysis. The price year was 2015 and the costs were reported in Great British Pound Sterling (£). The model structure was developed following a review of intervention trials [8] and cost-effectiveness analyses [15] and verified with a multi-disciplinary clinical team in Newham, East London, who were engaged in developing a Borough-wide diabetes prevention programme. The model comprised four health states (normoglycaemia, intermediate hyperglycaemia (either IFG, IGT or HbA1c), T2DM and death). The outcomes of the analysis were cost per QALY gained, where the QALYs were calculated using SF-6D utility values. We adopted a 50-year time horizon with annual cycles. Costs and utilities were discounted by an annual discount rate of 3.5% per year, which is the rate recommended by NICE [22].

Both deterministic and probabilistic models were evaluated; the probabilistic model was used to account for non-linearity and correlations in parameters and to characterise the decision uncertainty. Deterministic sensitivity analysis was undertaken to evaluate alternative scenarios where there are differences in definitions (e.g. American Diabetes Association or World Health Organization (WHO) diagnostic criteria) or primary clinical data is not available (e.g. long-term effect of interventions).

Three populations were evaluated in the model, namely individuals with IFG, IGT and HbA1c in the ‘at-risk’ range, across 12 different diagnosis-treatment pairs: IGT_pragmatic lifestyle, IGT_intensive lifestyle, IGT_metformin, IGT_no intervention, IFG_pragmatic lifestyle, IFG_intensive lifestyle, IFG_metformin, IFG_no intervention, HbA1c_pragmatic lifestyle, HbA1c_intensive lifestyle, HbA1c_metformin, and HbA1c_no intervention.

### Model structure

**Fig. 1:**
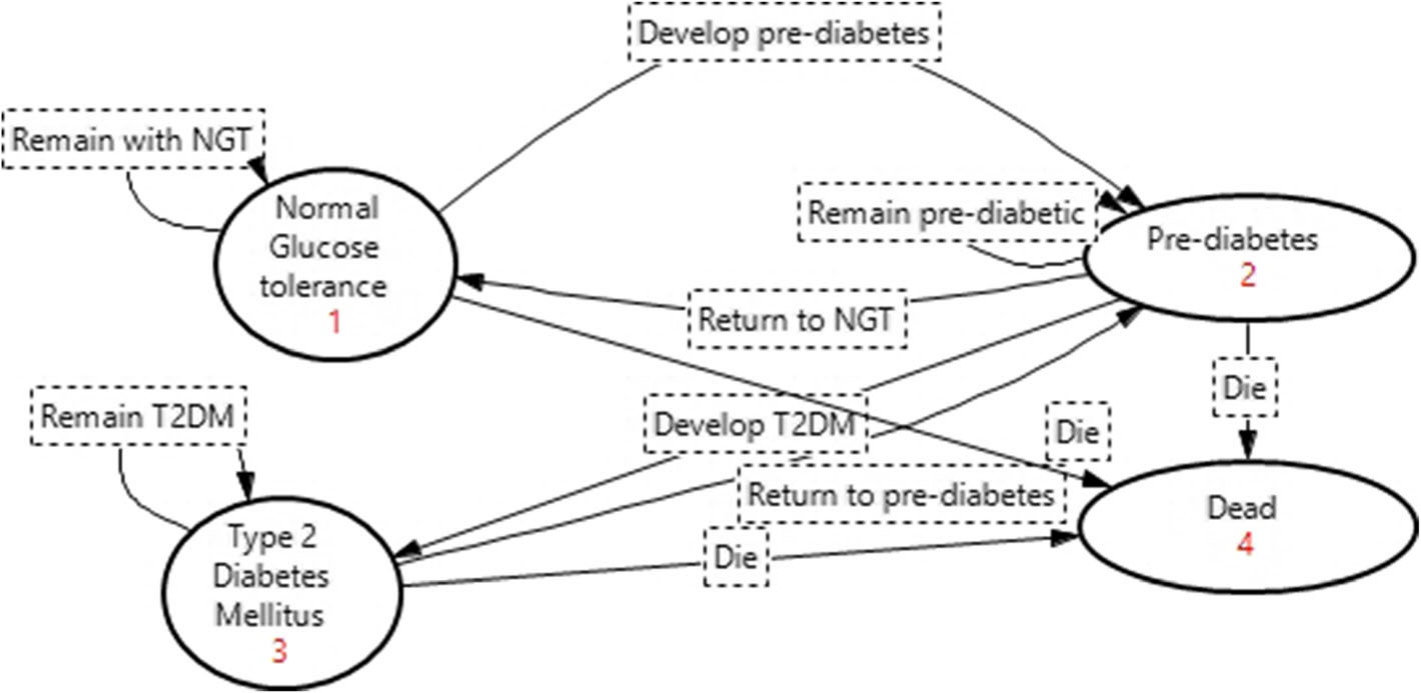
State transition diagram

We assumed that the population entered the model with a diagnosis of intermediate hyperglycaemia (IFG, IGT, HbA1c) and could transition to T2DM, normoglycaemia or death, with the probability of transitioning between states modified by the type of intervention the participant receives. Participants who were normoglycaemic could transition to intermediate hyperglycaemia or death, but not directly to T2DM. To reflect disease progression/clinical reality participants who transitioned to T2DM remained in this state until the end of the modelling period or death (Fig. 1).

For our population-level case study of England, we assumed all adults aged 50–59 years with diagnosed IFG, IGT or HbA1c would be offered an intervention, but that only 50% of the population with intermediate hyperglycaemia would be diagnosed and that 50% who were offered an intervention would fail to enrol. These assumptions match those utilised by NICE in the costing template for diabetes prevention guidance [23], as primary studies of enrolment and compliance in this area show a very wide range of participation rates [24]. We assumed that intermediate hyperglycaemia was diagnosed in one of two ways, namely (1) an incidental finding when blood tests were taken for another purpose or (2) through assessment of glycaemic status during an NHS Health Check England, a clinical assessment offered to all 40–74 year olds in England without pre-existing diabetes or cardiovascular disease (with coverage of 13.7–22.4% reported nationally in in the 50–59 year age group) [25].

### Model parameters

IFG, IGT and HbA1c are distinct physiological states and differ in terms of epidemiological parameters, cost of care and health utilities (Table 1). However, a single individual may have one, two or three types of intermediate glycaemia concurrently.

**Table 1:** Baseline population – key parameter values

Health state	Diagnostic test	Diagnostic criteria	Prevalence	Annual incidence of T2DM	Annual cost of care (£, 2015)	Utility (QALYs)
Normoglycaemia (NGT)	Any of fasting blood glucose, post-load glucose or glycated haemoglobin	Fasting glucose: <5.6 mmol/L Post-load glucose: <7.0 mmol/L HbA1c: <6.0 mmol/mol			£773 *[Gamma distribution, SE: £102.63]*	0.768 *[Beta distribution, SE: 0.10]*
Impaired fasting glucose (IFG)	Fasting blood glucose: blood glucose test after a period of fasting (typically overnight)	Fasting glucose: 5.6-6.9 mmol/L	Isolated IFG: 12.76% IFG + IGT: 1.49% IFG + HbA1c: 6.61% IFG + IGT + HbA1c: 1.06%	3.55% *[Beta distribution, SE: 0.006]*	£869 *[Gamma distribution, SE: 104.56]*	0.759 *[Beta distribution, SE: 0.11]*
Impaired glucose tolerance (IGT)	Post-load glucose: blood glucose test 2 hours after consuming a drink containing 75 g of sugar	2 hour post-load glucose: 7.0-11.1 mmol/L	Isolated IGT: 7.50% IGT + HbA1c: 3.31%	4.54% *[Beta distribution, SE: 0.004]*	£946 *[Gamma distribution, SE: 101.52]*	0.746 *[Beta distribution, SE: 0.10]*
HbA1c	Glycated haemoglobin: blood test which estimates blood glucose levels over previous 2-3 months	6.0-6.4 mmol/mol	Isolated HbA1c: 7.53%	3.56% *[Beta distribution, SE:0.017]*	£869 *[Gamma distribution, SE: 104.56]*	0.759 *[Beta distribution, SE: 0.11]*
Type 2 Diabetes Mellitus (T2DM)	Any of fasting blood glucose, post-load glucose or glycated haemoglobin	Fasting glucose: >6.9 mmol/l 2 hour post-load glucose: >11.1 mmol/L			£1,179-£2,939 Increasing linearly from Year 1-15 *[Gamma distribution, SE: 270.00]*	0.738 *[Beta distribution, SE: 0.12]*

Parametric form and standard error of distribution used in probabilistic sensitivity analysis in italics

Sources: Diagnostic criteria for HbA1c and IGT [26] and for IFG [27], prevalence [28], annual incidence type 2 Diabetes [10], costs [36–39], utilities [40]

#### Clinical and epidemiological parameters

Diagnostic criteria for prediabetes reflected those of the NHS Diabetes Prevention Programme [13], WHO diagnostic criteria for HbA1c and IGT [26], and American Diabetes Association criteria for IFG [27] (Table 1). Prevalence of IFG, IGT and at-risk HbA1c, as well as of the combinations of different types of intermediate hyperglycaemia, was extracted from a UK-based study [28] and the annual probability of transitioning to T2DM was obtained from a meta-analysis with different transition probabilities assumed for IFG, IGT and HbA1c [10]. All-cause age-standardised mortality rates were determined from the Office of National Statistics in England [29], with increased risk of death calculated for participants with intermediate hyperglycaemia or T2DM [30].

For IFG and IGT, relative risks of developing T2DM or reverting to normoglycaemia with lifestyle interventions were derived from meta-analyses [8, 15, 20]. Relative risks for metformin were drawn from the United States Diabetes Prevention Program Outcomes Study (USDPPOS) as this is the only long-term follow-up study of this intervention [31]. To our knowledge, there is only a single randomised controlled study (a sub-group analysis of the USDPP) [12] reporting relative risks of participants identified on the basis of HbA1c. Our model drew from this single analysis (in which participants also had IGT +/- IFG). We assumed that the reduction in risk related to metformin was constant over 15 years for participants with IGT and IFG and over 10 years for participants identified on the basis of HbA1c, as these were the longest periods of follow-up that have been published for each population [12, 31]. Based on a recent meta-analysis, we assumed that reduction in risk declined following cessation of the intensive lifestyle programme [7] and ceased 10 years after the intervention commenced. As no long term follow-up studies of pragmatic lifestyle programmes have been undertaken, we conservatively assumed the risk reduction persisted only for the duration of the intervention. Finally, we assumed that adherence was equivalent to that seen in the clinical trials from which relative risks were derived.

#### Interventions

The low-intensity lifestyle programme was based on NICE guidance [32] and includes a core component of 13 group education sessions in the first year followed by 7 maintenance sessions over the following 2 years, delivered by diabetes prevention facilitators, with annual review by a general practitioner and blood tests by a practice nurse. The high-intensity lifestyle programme was based on the USDPP [33], and includes 16 one-to-one education sessions delivered by a dietician and 4 exercise sessions supervised by a physiotherapist in the first year as well as 12 individual visits and 4 supervised exercise sessions in the second and third year. Further, it includes 1–2 reminder phone calls a month and annual clinical review and blood tests. In terms of metformin, a dosage of 850 mg twice a day was assumed, in line with the USDPP [33], with annual titration review and blood tests by a practice nurse and annual review by a general practitioner. The low-intensity lifestyle intervention lasted 2 years, the high-intensity lifestyle intervention lasted 3 years and we assumed that metformin therapy continued as long as the participant had intermediate hyperglycaemia. The base case of no intervention assumed that people with a diagnosis of intermediate hyperglycaemia received no additional treatment, as was the case in the majority of England before the commencement of the national pilots in diabetes prevention in 2017.

#### Costs (Additional file 1: Appendix 2)

We calculated the costs of lifestyle programmes by applying Personal Social Services Research Unit (PSSRU) staff cost estimates [34] to constituent activities described in publications regarding the USDPP [33] and NICE guidance [32], and using published estimates of diagnostic test costs [35]. We used the British National Formulary to calculate medication costs [36]. As an NHS perspective was adopted, we did not include indirect costs such as productivity loss or participants’ out-of-pocket costs.

Costs of T2DM were determined from a UK study of resource utilisation in diabetic care [37]. We assumed costs of diabetes increase linearly over 15 years from the time of diagnosis to reflect the increasing cost of diabetic complications over time, in line with the approach taken by NICE [23]. Costs of other health states were calculated as proportions of T2DM costs, derived from two European studies [38, 39]. All costs were inflated to 2015 values. Unrelated healthcare costs (not related to diabetes or its complications) that accrue due to prolonged life were not included in the base case, but were considered in sensitivity analysis.

#### Utilities

Utilities were measured in QALYs and were derived for each health state from a Swedish study that utilised SF-36 questionnaires, converting responses via the SF-6D index to utilities [40]. This is the only source of utilities, to our knowledge, that measured quality of life in IFG and IGT separately. Incremental utilities associated with each intervention were drawn from the USDPP [33], with both low- and high-intensity lifestyle programmes assumed to be associated with the same incremental utility.

Table 2 outlines the key parameter values, with Additional file 1: Appendix 1 outlining data sources, assumptions and limitations of these values.

**Table 2:** Interventions – key parameter values

Intervention	Annual cost of intervention (£, 2015)	Incremental utility associated with intervention (QALY)	Relative risk of developing T2DM
Pragmatic lifestyle programme	Yr 1: £203.44 Yr 2: £80.02	0.0189 *[Beta distribution, SE: 0.001]*	During intervention: IFG: 0.74/IGT: 0.74/HbA1c: 0.74 *[Lognormal distribution, SE: 0.11]*
Intensive lifestyle programme	Yr 1: £1225 Yr 2: £689 Yr 3: £671	0.0189 *[Beta distribution, SE: 0.001]*	During intervention: IFG: 0.63/IGT: 0.55/HbA1c: 0.71 *[Lognormal distribution, SE: 0.10 (IFG), 0.07 (IGT), 0.10 (HbA1c)]* Up to 7 years post- intervention: IFG: 0.80/IGT: 0.80/HbA1c: 0.71 *[Lognormal distribution, SE: 0.06 (IFG), 0.06 (IGT), 0.10 (HbA1c)]*
Metformin	£124.25	0.0031 *[Beta distribution, SE: 0.002]*	IFG: 0.82/IGT: 0.82/HbA1c: 0.62 *[Lognormal distribution, SE: 0.05(IGT), 0.05 (IFG), 0.10 (HbA1c)]*

Parametric form and standard error of distribution used in probabilistic sensitivity analysis in italics

Sources: Intervention costs (calculated – see Additional file 1: Appendix 2), incremental utilities [32], relative risks [8, 12, 15, 20, 21]

### Analyses

Two types of analyses were undertaken. Firstly, that of impact on an individual participant in a prevention programme, followed by impact of a nation-wide prevention programme, using England as a case study.

Analyses of individual participants included (1) discounted cumulative healthcare costs (including costs of diagnostic tests and primary and secondary care associated with the intervention, intermediate hyperglycaemia, T2DM and complications of T2DM), (2) discounted QALYs, (3) incidence of T2DM, (4) average number of years with T2DM, (5) cost-effectiveness ratios in £/QALY, and (6) incremental cost-effectiveness ratios (ICERs), in £/QALY (for non-dominated interventions). Individuals are frequently diagnosed with more than one type of intermediate hyperglycaemia (Table 1). All participants with each type of intermediate hyperglycaemia (alone or in combination with other types of intermediate hyperglycaemia) were analysed in each arm of the model. For example, the IGT arm includes participants with either IGT in isolation, IGT and IFG, IGT and HbA1c, or IGT, IFG and HbA1c in the at-risk range.

Analyses of a nation-wide prevention programme included (1) discounted annual incremental costs, (2) discounted cumulative incremental costs, (3) discounted incremental costs as a percentage of the total diabetes expenditure [17], and (4) cumulative incidence of T2DM. To account for individuals with multiple types of intermediate hyperglycaemia, the costs and effects in the IGT arm of the analysis was assumed to represent all individuals with a diagnosis of IGT (participants with IGT in isolation, IGT and IFG, IGT and HbA1c in the at-risk range, and IGT, IFG and HbA1c in the at-risk range), the costs and effects in the IFG arm of the analysis was assumed to represent all individuals with isolated IFG and with IFG and HbA1c in the at-risk range, and the costs and effects of the HbA1c arm of the analysis was assumed to represent all individuals with isolated HbA1c in the at-risk range.

### Sensitivity analyses

We assessed parameter uncertainty with (1) deterministic one-way sensitivity analysis, altering all parameter values by ±10%, (2) probabilistic sensitivity analysis and (3) deterministic scenario analyses where primary clinical data was not available to create a distribution (e.g. duration of intervention effect) or differences in clinical definitions existed (e.g. IFG diagnosed by WHO criteria).

### Validation

We validated the model in accordance with the AdVISHE (Assessment of the Validation Status of Health-Economic Decision Models) checklist [41] (Additional file 1: Appendix 6). Three experts tested the face validity of the model structure, inputs and outputs, and their suggestions were incorporated in the final model. Extreme value testing and audit of Markov cohort traces was undertaken by the authors and the structure of formulae were reviewed in a session with the TreeAge support team. Model outputs were validated against empirical data, including mortality data for England and estimates of current prevalence of T2DM by age group.

## Results

### Outcomes for individual participants in a prevention programme

The base case results of deterministic sensitivity analysis are presented in Tables 3, 4, and 5. In participants with all types of intermediate hyperglycaemia, pragmatic lifestyle programmes, intensive lifestyle programmes and metformin all increased costs, improved QALYs and reduced diabetes incidence compared with no intervention.

**Table 3:** Costs and consequences for individual participants in a prevention programme

Method of identifying participants	Intervention	Total cost (£,2015)	Total QALYs	Prevalence of T2DM after 50 years (%)	Average number of years lived with T2DM after 50 years
IGT	No intervention	17,772	11.53	42%	5.75
	Pragmatic lifestyle programme	17,774	11.59	41%	5.43
	Intensive lifestyle programme	18,423	11.76	33%	3.97
	Metformin	17,988	11.60	38%	5.03
IFG	No intervention	17,429	12.13	38%	5.34
	Pragmatic lifestyle programme	17,440	12.19	37%	5.07
	Intensive lifestyle programme	18,452	12.28	31%	3.98
	Metformin	17,908	12.20	35%	4.68
HbA1c	No intervention	17,436	12.13	38%	5.35
	Pragmatic lifestyle programme	17,446	12.19	37%	5.08
	Intensive lifestyle programme	18,507	12.27	31%	4.03
	Metformin	17,475	12.23	33%	4.18

Differences between costs and QALYs of ‘no intervention’ in the groups with IGT, IFG and HbA1c are due to: 1) higher hazard ratios of death with IGT relative to IFG/HbA1c, ii) lower baseline utilities for IGT relative to IFG/HbA1c and iii) higher baseline transition probabilities to T2DM with IGT relative to IFG/HbA1c, as outlined in Additional file 1: Appendix 1

**Table 4:** Diabetes incidence and risk reduction over 10 years and 50 years

Method of identifying participants	No intervention		Low intensity lifestyle programme				High intensity lifestyle programme				Metformin			
	Incidence T2DM		Incidence T2DM		Relative risk reduction		Incidence T2DM		Relative risk reduction		Incidence T2DM		Relative risk reduction	
	After 10 years	After 50 years	After 10 years	After 50 years	At 10 years	At 50 years	After 10 years	After 50 years	At 10 years	At 50 years	After 10 years	After 50 years	At 10 years	At 50 years
IGT	23%	42%	22%	41%	7%	3%	14%	33%	39%	21%	20%	38%	16%	9%
IFG	19%	38%	18%	37%	7%	3%	13%	31%	35%	17%	16%	35%	16%	9%
HbA1c	19%	38%	18%	37%	7%	3%	13%	31%	35%	17%	13%	33%	35%	14%

**Table 5:** Incremental cost effectiveness ratios and cost-effectiveness relative to no intervention for individual participants in a prevention programme

Method of identifying participants	Intervention	Incremental cost effectiveness ratio (ICER) *(relative to next best intervention)*				Cost effectiveness ratio (CER) *(relative to no intervention)*			
		Incremental cost (£, 2015)	Incremental effect (QALYs)	ICER (£/QALY)	Probability ICER < £20,000/QALY	Cost vs no intervention (£, 2015)	Effect vs no intervention (QALYs)	CER (£/QALY)	Probability CER < £20,000/QALY
IGT	No intervention	-	-	-	-	-	-	-	-
	Low-intensity lifestyle	3	0.06	44	98.19%	3	0.06	44.33	98.19%
	Metformin	Subject to extended dominance				367	0.07	5,224	75.86%
	High-intensity lifestyle	649	0.18	3,707	74.58%	652	0.23	2,775	80.5%
IFG	No intervention	-	-	-	-	-	-	-	-
	Low-intensity lifestyle	11	0.06	195	98.5%	11	0.06	195	98.5%
	Metformin	Subject to extended dominance				479	0.07	6,842	76.28%
	High-intensity lifestyle	1,012	0.09	11,219	75.09%	1,023	0.15	6,820	81.44%
HbA1c	No intervention	-	-	-	-	-	-	-	-
	Low-intensity lifestyle	11	0.06	186	97.79%	11	0.06	186	97.79%
	Metformin	28	0.05	600	50.40%	39	0.10	372	77.89%
	High-intensity lifestyle	1,032	0.04	25,481	40.38%	1,071	0.15	7,376	71.28%

#### Incremental cost-effectiveness ratios (ICERs) – comparison with the next best alternative

**Fig. 2:**
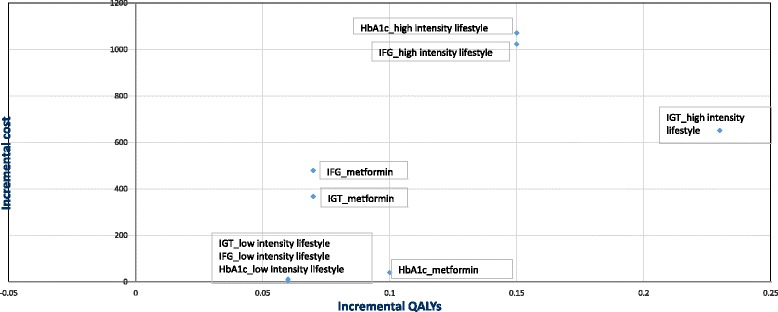
Cost-effectiveness plane: incremental cost and QALYs relative to no intervention

For all three populations, the low-intensity lifestyle programme was the most cost-effective option, with ICERs of £44/QALY, £195/QALY and £186/QALY in populations with IGT, IFG and HbA1c in the at-risk range, respectively. At the current NICE willingness-to-pay threshold of £20,000/QALY, intensive lifestyle interventions were cost-effective relative to the next best alternative (low-intensity lifestyle programme), with ICERs of £3707 and £11,219 for IGT and IFG, respectively. For the population with HbA1c in the at-risk range, metformin was also found to be cost-effective relative to the next best alternative (low-intensity lifestyle programmes), with an ICER of £600/QALY; this was the only population for which metformin was not extendedly dominated (a combination of pragmatic and intensive lifestyle interventions was not more cost-effective than metformin) (Table 5, Fig. 2). However, due to effect sizes in participants with HbA1c being derived from a single clinical study, results for this population should be treated cautiously. At a willingness-to-pay threshold of £20,000/QALY, the probability of being cost-effective relative to the next best alternative was 98%, 99% and 98% for low-intensity lifestyle programmes and 75%, 75% and 40% for high-intensity lifestyle programmes for participants with IGT, IFG and HbA1c, respectively. The probability that metformin was cost-effective relative to the next best alternative was 50% for participants with HbA1c (Additional file 1: Appendix 5).

#### Cost-effectiveness ratios – comparison with no intervention

Compared to no intervention, the low-intensity lifestyle programme was the most cost-effective option with cost-effectiveness ratios of £44/QALY, £195/QALY and £186/QALY in populations with IGT, IFG and HbA1c in the at-risk range, respectively. Cost-effectiveness of intensive lifestyle interventions were £2775/QALY, £6820/QALY and £7376/QALY and of metformin were £5224/QALY, £6842/QALY and £372/QALY relative to no intervention for IGT, IFG and HbA1c, respectively (Table 5, Fig. 2). At a willingness-to-pay threshold of £20,000/QALY, the probability of being cost-effective was 98%, 99% and 98% for low-intensity lifestyle programmes, 81%, 81% and 71% for high-intensity lifestyle programmes, and 76%, 76% and 78% for metformin in participants with IGT, IFG and HbA1c, respectively (Additional file 1: Appendix 5).

#### Effect on diabetes prevalence

With no intervention, 42% of the IGT population and 38% of the IFG and HbA1c population developed T2DM over 50 years. Diabetes incidence was reduced to 41%, 33% and 38% in the IGT population, 37%, 31% and 35% in the IFG population, and 37%, 31% and 33% in the HbA1c population with pragmatic lifestyle programmes, intensive lifestyle programmes and metformin, respectively (Table 4).

### Outcomes of a nation-wide prevention programme

Incident cases of T2DM would be reduced by 0.3–1.5% over 50 years in those aged 50–59 years if a pragmatic lifestyle programme was offered to everyone with a diagnosis of either IFG, IGT or HbA1c in the at-risk range in this age group in England (Table 6). A national intensive lifestyle programme would lead to the greatest population health benefits, with a 1.9–3.1% reduction in diabetes incidence and 2.7–3.4% reduction in the number of years with T2DM. The type of prediabetes has a significant impact on population-level outcomes due to the substantially higher prevalence of IFG and high HbA1c than IGT.

**Table 6:** Outcomes for an England-wide prevention programme

Method of identifying participants	Intervention	Reduction in incident cases of T2DM (number of cases)	% reduction in incident cases of T2DM	Reduction in average number of years lived with T2DM (number of years)	% reduction in average number of years lived with T2DM (%)
IGT	Pragmatic lifestyle programme	2,938	0.3%	0.03	0.5%
	Intensive lifestyle programme	20,494	1.9%	0.15	2.7%
	Metformin	9,388	1.9%	0.07	1.3%
IFG	Pragmatic lifestyle programme	11,582	1.1%	0.04	0.7%
	Intensive lifestyle programme	32,119	3.0%	0.19	3.4%
	Metformin	20,863	1.9%	0.11	2.0%
HbA1c	Pragmatic lifestyle programme	15,856	1.5%	o.03	0.6%
	Intensive lifestyle programme	33,027	3.1%	0.15	2.8%
	Metformin	29,163	2.7%	0.14	2.5%

**Fig. 3:**
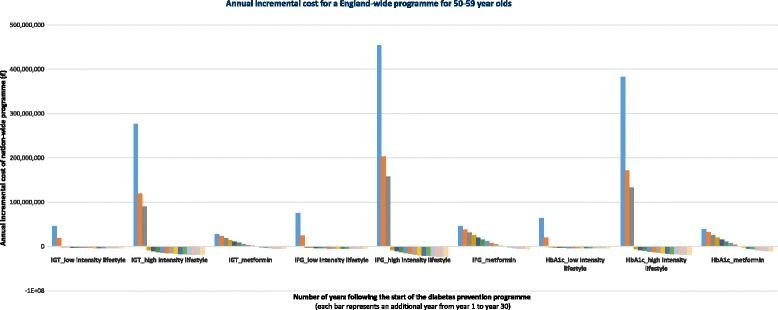
Annual incremental costs of an England-wide programme

**Fig. 4:**
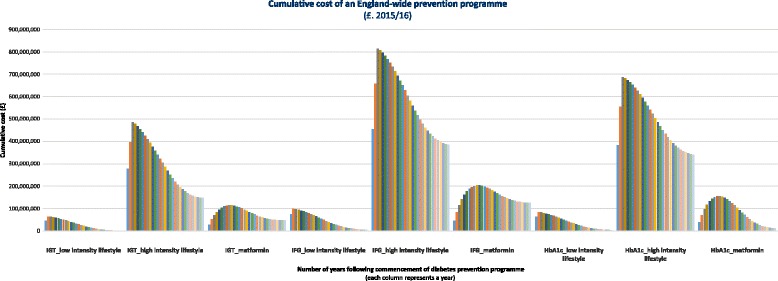
Cumulative cost of an England-wide programe

Annual incremental costs are negative from year 3 for pragmatic lifestyle programmes, from year 4 for intensive lifestyle programmes and from year 10 for metformin, relative to no intervention (Fig. 3). Cumulative costs remain positive over the 50-year modelling period relative to no intervention (Fig. 4). Assuming no existing diabetes services are displaced, an England-wide prevention programme requires an investment (as a percentage of total diabetes costs) of 0.5–0.9% in year 1 and 0.2–0.3% in year 2 for a pragmatic lifestyle intervention, and 3.1–5.2% in year 1, 1.4–2.3% in year 2 and 1.0–1.8% in year 3 for an intensive lifestyle programme, depending on the type of participants targeted (Additional file 1: Appendix 3).

### Sensitivity analysis

Key factors impacting cost-effectiveness calculations in one-way sensitivity analysis were health state utilities, hazard ratios of death, relative risks of T2DM and costs of the interventions. Additional scenarios examining extended duration of intervention effect, use of WHO criteria to diagnose IFG, increased/decreased intervention costs and inclusion of unrelated healthcare costs (Additional file 1: Appendix 4) resulted in differences from the base case analysis. Firstly, pragmatic lifestyle programmes are cost-saving in all participants when intervention effect is extended. Secondly, pragmatic lifestyle programmes are cost saving if the WHO criteria are used to diagnose IFG, with reduced budget impact at a population level but fewer cases of T2DM prevented. Thirdly, metformin is cost saving in participants with HbA1c when intervention effect is extended. Finally, intensive lifestyle programmes are cost-effective in participants with HbA1c when intervention costs were decreased by 20%. All interventions remained cost-effective relative to no intervention when unrelated healthcare costs were included in the analysis.

## Discussion

### Principal findings

This study has produced six major findings. Firstly, low-intensity lifestyle interventions are the lowest cost diabetes prevention programme over a participant’s lifetime in all types of intermediate hyperglycaemia. Secondly, high-intensity lifestyle interventions deliver the greatest health benefit in terms of reducing diabetes incidence, years lived with T2DM and QALYs gained in participants with all types of intermediate hyperglycaemia. Thirdly, at a population-level, the type of intervention has the greatest impact on costs while the type of intermediate hyperglycaemia used for inclusion in prevention programmes has the greatest impact on percentage reduction in incident cases. Fourthly, low- and high-intensity lifestyle programmes are very cost-effective in participants with IFG and IGT, while metformin is not a cost-effective option in these populations; these results were consistent across a range of parameter values. Fifthly, while budget impact as a percentage of total diabetes expenditure is small, these interventions require a net increase in diabetes expenditure (assuming existing services are not displaced) over 2, 3 and 9 years in the case of low-intensity lifestyle, high-intensity lifestyle and metformin, respectively. Subsequent savings due to reduced incidence of T2DM are insufficient to entirely offset this increased expenditure. Finally, impact on incidence of T2DM at a population level is small due to the lack of overlap between different types of intermediate hyperglycaemia and issues with participation in screening tests, adherence to interventions and attenuation in the treatment effect over time.

This study’s results are comparable with previously published economic evaluations of diabetes prevention programmes, which found ICERs ranging from cost saving to £134,420/QALY with a median value of £7490/QALY for lifestyle programmes, and ranging from cost saving to £32,430/QALY with a median value of £8428/QALY for metformin [15] in comparison to no intervention. Differences in assumptions regarding intervention cost and effect and uncertainty regarding key parameter values (e.g. duration of intervention effect), account for the range of ICERs in published economic evaluations.

### Implications for policymakers

This study provides a quantification of a number of key tensions in diabetes prevention policy, including (1) whether to select participants for which interventions will be the most cost-effective (those with IGT) or participants identified by tests that are widely used in current clinical practice (those with high HbA1c or IFG), (2) whether to target interventions at populations with the most attractive ICERs (those with IGT) or populations in which the greatest population-wide impact could be achieved (those with IFG according to American Diabetes Association criteria), and (3) whether to minimise budget impact by providing low-intensity lifestyle programmes or maximise reduction in diabetes incidence and QALYs gained by providing high-intensity lifestyle programmes.

On balance, this analysis suggests that current English national policy of targeting prevention programmes at participants with IFG or HbA1c, and not recommending metformin as first-line prevention, will be cost-effective and have the most favourable budget impact. However, the modest reduction in incidence of T2DM importantly suggests that this approach will be insufficient to address the substantial growth in diabetes forecast for the coming decades. Therefore, the search for additional interventions should continue.

We did not formally evaluate costs and effects in other countries. However, effect sizes in this model are drawn from international studies, and therefore our conclusions regarding gains in QALYs, reduction in incidence of T2DM and years with T2DM should be broadly generalisable, assuming equivalent prevalence of intermediate hyperglycaemia.

### Strengths and limitations

This study adds to previous economic evaluations by quantifying the impact of different types of intermediate hyperglycaemia and different intensities of lifestyle programme as well as by estimating costs and consequences at an individual participant level and a national programme level, using a case study of England. This study’s limitations include the availability of primary clinical data and the structure and scope of the Markov model. In terms of data availability, there were limited primary clinical data from trials to model participants with intermediate hyperglycaemia identified by HbA1c, quantify the long-term effect of pragmatic lifestyle interventions, differentiate the reduction in diabetes incidence due to low-intensity lifestyle interventions by type of intermediate hyperglycaemia, or evaluate the long-term effects of metformin in isolation by age group, since the USDPP Outcomes Study data used in this analysis relates to a cohort that received lifestyle advice in addition to metformin from year 4 of the 10-year intervention. Another major shortcoming is the absence of evidence on the impact of lifestyle on endpoints important to patients such as, for example, the complication of diabetes and death. In terms of the model structure, we elected to use a Markov model to compare our findings with those of previous economic evaluations, the majority of which use Markov models [15]. However, the underlying physiological changes in intermediate hyperglycaemia and diabetes are continuous variables (fasting glucose, post-load glucose or HbA1c), which are better suited to simulation modelling. In addition, simulation modelling requires more detailed data which were not available for all types of participants and interventions modelled. In terms of the scope of the model, we modelled only costs and QALYs relating to diabetes and its complications, whereas interventions may have beneficial effects on other types of disease (e.g. obesity-related cancers, dementia) that are not captured, but would likely improve the cost-effectiveness of lifestyle programmes. In addition, we did not explicitly model adverse effects of metformin, which we assumed were accounted for in the lower incremental utilities associated with metformin relative to lifestyle programmes.

### Suggestions for future research

This study has confirmed five areas where further research would be beneficial. Firstly, evaluating the effect of lifestyle programmes and metformin in participants identified on the basis of HbA1c. Secondly, undertaking longer term follow-up of pragmatic lifestyle programmes to evaluate the duration and profile of the reduction in risks of T2DM. Thirdly, evaluating the impact of lifestyle programmes on the complications of T2DM, including death. Fourthly, modelling the effects of diabetes prevention programmes on other obesity-related diseases. Finally, consideration of the role of broader social and environmental programmes (e.g. sugar tax, changes to the physical environment) on diabetes incidence as, based on the findings of this study, individual lifestyle programmes and metformin are unlikely to be sufficient to address the vast majority of incident cases of T2DM.

## Conclusions

Different categories of intermediate hyperglycaemia and varying intensities of lifestyle intervention do lead to differences in the cost-effectiveness of diabetes prevention programmes. Low- and high-intensity lifestyle programmes are cost-effective in participants with IFG or IGT. Metformin appears cost-effective in populations with HbA1c in the at-risk range; however, these results should be treated cautiously due to the lack of primary clinical data on the effects of prevention programmes in participants with isolated high HbA1c. No single option has the most attractive cost-effectiveness profile, budget impact and impact on incident cases of T2DM or years with T2DM, with prevention policy facing a trade-off between these factors.

## Additional file

Additional file 1: Economic evaluation of type 2 diabetes prevention programmes: does the type of pre-diabetes and intensity of intervention matter? Appendices [42, 43]. (DOCX 3780 kb)

## Abbreviations

HbA1c: glycated haemoglobin
IFG: impaired fasting glucose
IGT: impaired glucose tolerance
NHS: National Health Service
NICE: National Institute of Clinical Excellence
PSSRU: Personal Social Services Research Unit
T2DM: type 2 diabetes mellitus
USDPP: United States Diabetes Prevention Program
WHO: World Health Organization.
