# Supplementary material for: Economic evaluation of type 2 diabetes prevention programmes: Markov model of low- and high-intensity lifestyle programmes and metformin in participants with different categories of intermediate hyperglycaemia
Source: BMC Med. 2018 Jan 30;16:16. doi: 10.1186/s12916-017-0984-4 (PMC5798197; doi:10.1186/s12916-017-0984-4)
Supplement: Additional file 1: — Economic evaluation of type 2 diabetes prevention programmes: does the type of pre-diabetes and intensity of intervention matter? Appendices [42, 43]. (DOCX 3780 kb) [file 12916_2017_984_MOESM1_ESM.docx]

**ECONOMIC EVALUATION OF TYPE 2 DIABETES PREVENTION PROGRAMMES: APPENDIX**

**Appendix 1: Parameter values, assumptions and limitations**

| **Description** | **Value** | **Source** | **Comments** |
| --- | --- | --- | --- |
| Cost of impaired glucose tolerance with no treatment | £ 946.00 | 1. Bachle C. Direct costs in glucose regulation: results from the population-based Heinz Neixdorf recall study. 2016. BMJ Open Diabetes Research and Care. 2. Gregory A. Nichols, Bhakti Arondekar and William H. Herman. Medical Care Costs One Year after Identification of Hyperglycemia below the Threshold for Diabetes Author. 2008. Medical Care ; 46 (3):287-292 | IFG costs are 73% of T2DM costs, NGT costs are 66% of T2DM costs. According to Nichols et al, IGT costs are 17% greater than IFG costs. Assume costs of HbA1c in the at risk range are equal to those of IFG. Use costs of uncomplicated T2DM for calculations. |
| Cost of impaired fasting glucose and HbA1c in the at risk range with no treatment | £ 869.00 |  |  |
| Cost of normal glucose tolerance | £ 773.00 |  |  |
| Cost of Type 2 Diabetes | £ 2,939.00 | 1. Hex N, Bartlett C, Wright Det al. Estimating the current and future costs of type 1 and type 2 diabetes in the United Kingdom, including direct health costs and indirect societal and productivity costs. 2012. Diabetic Medicine 29: 855–862 2. National Institute of Clinical Excellence. Preventing type 2 diabetes: risk identification and interventions for individuals at high risk. Costing template. July 2012 | Increasing linearly over 15 years from cost of IGT to cost of T2DM with complic1ations (£2,939) based on assumptions used in NICE costing template. |
| Cost of pragmatic lifestyle intervention Year 1 | £ 203.44 | 1. Curtis B, Burns A. Unit Costs of Health and Social Care 2015. Personal Social Services Research Unit 2. National Institute for Health and Care Excellence. Type 2 diabetes: prevention in people at high risk. NICE Guideline PH 38. 2012 | PSSRU unit costs applied to activities outlined as part of recommended lifestyle programme in NICE guidance |
| Cost of pragmatic lifestyle interventions year 2 | £ 80.02 |  |  |
| Cost of implementing the USDPP in the UK in 2015 – Year 1 | £ 1,225 | 1. Curtis B, Burns A. Unit Costs of Health and Social Care 2015. Personal Social Services Research Unit 2. Herman WH, Hoerger TJ, Brandle M, Hicks K, Sorensen S, Zhang P, et al, Diabetes Prevention Programme Research Group. The cost-effectiveness of lifestyle modification or metformin in preventing type 2 diabetes in adults with impaired glucose tolerance. Ann Intern Med. 2005; 142:323-32 | PSSRU unit costs applied to activities outlined as part of USDPP |
| Cost of Year 2 of USDPP in 2015 UK | £ 689 |  |  |
| Cost of Year 3 of USDPP | £ 671 |  |  |
| Cost of metformin | £ 124.25 | 1. Herman WH, Hoerger TJ, Brandle M, Hicks K, Sorensen S, Zhang P, et al, Diabetes Prevention Programme Research Group. The cost-effectiveness of lifestyle modification or metformin in preventing type 2 diabetes in adults with impaired glucose tolerance. Ann Intern Med. 2005; 142:323-32 2. Khunti K, Gillies CL, Taub NA, Mostafa SA, Hiles SL, Abrams KR, Davies MJ. A comparison of cost per case detected of screening strategies for Type 2 diabetes and impaired glucose regulation: modelling study. Diabetes Res Clin Pract. 2012 Sep;97(3):505-13 | Calculated based on BNF data, blood tests costs (Khunti, 2010) and GP review based on a modified version of USDPP metformin intervention (Herman, 2005) |
| Annual discount rate for costs | 0.035 | National Institute of Clinical Excellence. Guide to the Technology Appraisal Process. |  |
| Discount rate for effectiveness | 0.035 |  |  |
| Hazard ratio: death in participants with IGT | 1.5 | DECODE Study Group. Is the current definition for diabetes relevant to mortality risk from all causes and cardiovsacular and noncardiovascular disease? Diabetes Care 2003 Mar; 26(3):688-696 | Hazard ratios applied to mortality rates derived from age- and sex- standardised mortality rates from the Office of National Statistics.  Assume mortality rate for individuals with HbA1c in the at risk range is equivalent to the mortality rate for individuals with IFG. |
| Relative risk of death with T2DM following IGT | 1.9 |  |  |
| Hazard ratio: death in participants with IFG or HbA1c in at risk range | 1.1 |  |  |
| Relative risk of death with T2DM following IFG or HbA1c in at risk range | 1.6 |  |  |
| Probability someone with IGT develops T2DM | 0.04546 | Morris D, Khunti K, Achana F, Srinivasan B, Gray L, Davies M, et al. Progression rates from HbA1c 6.0–6.4% and other prediabetes definitions to type 2 diabetes: a meta-analysis. Diabetologia. 2013;56(7):1489-93 |  |
| Probability someone with IFG (ADA criteria) develops T2DM | 0.0474 |  |  |
| Probability someone with HbA1c in at risk range develops T2DM1 | 0.0355 |  |  |
| Probability someone with IGT returns to NGT with no treatment | 0.08969 | Balk EM, Earley A, Raman G, et al. Combined diet and physical activity promotion programs to prevent type 2 diabetes among persons at increased risk: a systematic review for the Community Preventive Services Task Force. Annals of internal medicine 2015;163(6):437-51 | No studies of participants with IFG or HbA1c in the at risk range used in meta-analysis. Therefore assume incidence of normoglycaemia in participant with IFG or high HbA1c is equal to incidence of normoglycaemia in participant with IGT |
| Probability someone with IFG or HbA1c returns to NGT with no treatment | 0.08969 |  |  |
| Probability someone with NGT develops IGT with no treatment | 0.0633 | Meigs JB, Muller DC, Nathan DM, Blake DR, Anders R. The Natural History of Progression From Normal Glucose Tolerance to Type 2 Diabetes in the Baltimore Longitudinal Study of Aging. Diabetes. 2003; 52: 1475-1484 |  |
| Probability someone with NGT develops IFG or ‘at risk’ HbA1c with no treatment | 0.0686 |  | Assume annual incidence of intermediate hyperglycaemia is equal for IFG and ‘at risk’ HbA1c |
| Relative risk of T2DM in participant undertaking pragmatic lifestyle program in years 1 and 2 | 0.74 | Ashra NB, Spong R, Carter P, et al. A systematic review and meta- analysis assessing the effectiveness of pragmatic lifestyle interventions for the prevention of type 2 diabetes mellitus in routine practice. London: Public Health England, 2015 | Meta-analysis of RCTs. Assume relative risk reduction equal for IFG, IGT and HbA1c as insufficient primary studies to analyse differences. |
| Relative risk of T2DM in someone with IGT undergoing an intensive lifestyle intervention years 1-3 | 0.55 | Roberts S, Barry E, Airoldi A, Craig D, Bevan G, Greenhalgh T. Preventing type 2 diabetes: systematic review of studies of cost-effectiveness of lifestyle programmes and metformin, with and without screening, for prediabetes. Submitted for publication | Meta-analysis of RCTs |
| Relative risk of T2DM in someone with IFG undergoing an intensive lifestyle intervention years 1-3 | 0.63 | Roberts S, Barry E, Airoldi A, Craig D, Bevan G, Greenhalgh T. Preventing type 2 diabetes: systematic review of studies of cost-effectiveness of lifestyle programmes and metformin, with and without screening, for prediabetes. Submitted for publication | Meta-analysis of RCTs |
| Relative risk of T2DM in someone with HbA1c undergoing an intensive lifestyle intervention years 1-3 | 0.71 | Diabetes Prevention Program Research Group. HbA1c as a Predictor of Diabetes and as an Outcome in the Diabetes Prevention Program: A Randomized Clinical Trial. Diabetes Care. 2014. Suppl. DOI: 10.2337/dc14-0886 | Subgroup analysis of USDPP. Participants had IGT +/- IFG in addition to HbA1c in ‘at risk’ range |
| Relative risk of T2DM in years 4-10 following cessation of intensive lifestyle intervention | 0.80 | Barry E, Roberts S, Oke J, Vijayaraghavan S, Normansell R, Greenhalgh TCan type 2 diabetes be prevented using screen-and-treat policies? Systematic review and meta-analysis of screening tests and interventions for pre-diabetes. BMJ. 2017 4;356:i6538 | Meta-analysis of RCTs. Assume relative risk reduction equal for IFG, IGT and HbA1c as insufficient primary studies in participants with IFG and HbA1c in at risk range to analyse differences. |
| Relative risk of T2DM in participants with IGT or IFG taking metformin | 0.82 | Diabetes Prevention Program Research Group. Long-term effects of lifestyle intervention or metformin on diabetes development and microvascular complications over 15-year follow-up: the Diabetes Prevention Program Outcomes Study. The Lancet Diabetes & Endocrinology. 2015;3(11):866-75 | Assume relative risk reduction equal for IFG and IGT as insufficient primary studies in participants with IFG to analyse differences. |
| Relative risk of T2DM in someone with HbA1c taking metformin | 0.62 | Diabetes Prevention Program Research Group. HbA1c as a Predictor of Diabetes and as an Outcome in the Diabetes Prevention Program: A Randomized Clinical Trial. Diabetes Care. 2014. Suppl. DOI: 10.2337/dc14-0886 | Subgroup analysis of USDPP. Participants had IGT +/- IFG in addition to HbA1c in ‘at risk’ range |
| Relative risk of NGT in people with IGT, IFG and HbA1c in at risk range receiving pragmatic lifestyle intervention | 1.4 | Vermunt PWA, Milder IEJ, Wielaard F, et al. A lifestyle intervention to reduce type 2 diabetes risk in dutch primary care: 2.5-year results of a randomized controlled trial. Diabetic Medicine. 2012;29(8):e223-e231 | Single study - the only RCT included in Ashra's meta-analysis that reported reversion to normoglycaemia for intervention and control |
| Relative risk of NGT in people with IGT, IFG and HbA1c receiving intensive lifestyle intervention | 1.53 | Balk EM, Earley A, Raman G, et al. Combined diet and physical activity promotion programs to prevent type 2 diabetes among persons at increased risk: a systematic review for the Community Preventive Services Task Force. Annals of internal medicine 2015;163(6):437-51 | Meta-analysis of RCTs. Assume relative risk reduction equal for IFG, IGT and HbA1c in at risk range as insufficient primary studies in participants with IFG and HbA1c in at risk range to analyse differences. |
| Relative risk that a participant with IGT, IFG and HbA1c would become normoglycemic on therapy with metformin | 1.1 | Knowler WC, Barrett-Connor E, Fowler SE, Hamman RF, Lachin JM, Walker EA, et al, Diabetes Prevention Programmeme Research Group. Reduction in the incidence of type 2 diabetes with lifestyle intervention or metformin. N Engl J Med. 2002; 346:393-403 | Single study – USDPP. Meta-analysis of RCTs. Assume relative risk reduction equal for IFG, IGT and HbA1c in at risk range as insufficient primary studies in participants with IFG and HbA1c in at risk range to analyse differences. |
| Additional utility per annum for people attending pragmatic lifestyle programs | 0.0189 | Herman WH, Hoerger TJ, Brandle M, Hicks K, Sorensen S, Zhang P, et al, Diabetes Prevention Programme Research Group. The cost-effectiveness of lifestyle modification or metformin in preventing type 2 diabetes in adults with impaired glucose tolerance. Ann Intern Med. 2005; 142:323-32 | Single study - USDPP. Assume QALY gain in pragmatic lifestyle programme is equal to that of intensive lifestyle programme |
| Additional utility from intensive lifestyle intervention (QALYs) | 0.0189 |  |  |
| Additional utility associated with taking metformin (QALYs) | 0.0031 |  |  |
| Utility of impaired glucose tolerance | 0.746 | Neumann A, Schoffer O, Norström F, Norberg M, Klug SJ and Lindholm L. Health-related quality of life for pre-diabetic states and type 2 diabetes mellitus: a cross-sectional study in Västerbotten Sweden. 2014. Health and Quality of Life Outcomes 2014; 12:150 | Single study in Sweden. Only study that reported QALYs for NGT, IGT, IFG and T2DM. Assume utility of individual with HbA1c in at risk range is equivalent to that of an individual with IFG. |
| Utility of impaired fasting glucose and HbA1c in at risk range (QALYs) | 0.759 |  |  |
| Utility of normoglycaemia (QALYs) | 0.768 |  |  |
| Utility of T2DM (QALYs) | 0.738 |  |  |
| Probability that someone with prediabetes would not enrol in diabetes prevention programme | 50% | 1. National Institute of Clinical Excellence. Preventing type 2 diabetes: risk identification and interventions for individuals at high risk. Costing template. July 2012. 2. Aziz Z, Absetz P, Oldroyd J, Pronk NP, Oldenburg B. A systematic review of real-world diabetes prevention programs: learnings from the last 15 years Implementation Science 2015 10:172 | NICE costing template assumes 50% of people with pre-diabetes are corectly identified and 50% enrol in an intervention. Aziz showed a wide range of enrollment in their review (0.28%-100%) |
| Probability someone with IFG/IGT would not be diagnosed with these conditions | 50% |  |  |

**Appendix 2: Costs of interventions**

**2.1. Pragmatic lifestyle programme:** An additional cost was allocated to blood tests

| **ACTIVITY** | **STAFF TYPE** | **YEAR 1** | | | | **YEAR 2** | | | |
| --- | --- | --- | --- | --- | --- | --- | --- | --- | --- |
|  |  | **Contact frequency** | **Time per contact (hrs)** | **Number of participants in group** | **Number of staff per group** | **Contact frequency** | **Time per contact (hrs)** | **Number of participants in group** | **Number of staff per group** |
| **Baseline history and physical examination** | GP | 1 | 0.50 | - | - | 1 | 0.18 | - | - |
| **Blood tests** | Practice nurse | - | - | - | - | 1 | 0.17 | - | - |
| **Group lifestyle sessions** | Support worker | 16 | 1.20 | 10 | 2 | - | - | - | - |
| **Maintenance group lifestyle sessions** | Support worker | 3 | 1.20 | 10 | 2 | 4 | 1.20 | 10 | 2 |

**2.2. Intensive lifestyle programme:** An additional cost was allocated to the course material and toolbox and blood tests

| **ACTIVITY** | **STAFF TYPE** | **YEAR 1** | | **YEAR 2** | | **YEAR 3** | |
| --- | --- | --- | --- | --- | --- | --- | --- |
|  |  | **Frequency per year** | **Time per contact (hrs)** | **Frequency per year** | **Time per contact (hrs)** | **Frequency per year** | **Time per contact (hrs)** |
| **Baseline history and physical examination** | GP | 1 | 1 |  |  |  |  |
| **Annual nurse review and blood tests** | Practice nurse |  |  | 1 | 0.33 | 1 | 0.33 |
| **Core curriculum** | Dietician | 16 | 1 |  |  |  |  |
| **Supervised activity session** | Dietician | 2.562 | 1 | 2.562 | 1 | 2.562 | 1 |
|  | Physiotherapist | 1.708 | 1 | 1.708 | 1 | 1.708 | 1 |
| **Lifestyle group sessions** | Dietician | 0.36 | 1.25 | 0.72 | 1.25 | 0.72 | 1.25 |
| **In-person visits** | Dietician or physiotherapist | 7.65 | 0.58 | 12.33 | 0.67 | 12.33 | 0.58 |
| **Phonecalls** | Support worker | 2.32 | 0.25 | 2.66 | 0.25 | 2.66 | 0.25 |
| **Reminder phone calls** | Support worker | 29.41 | 0.08 | 17.45 | 0.08 | 17.45 | 0.08 |

**2.3. Metformin:** An additional cost was allocated for blood tests and medication

| **ACTIVITY** | **STAFF TYPE** | **ANNUAL** | |
| --- | --- | --- | --- |
|  |  | **Frequency per year** | **Time per contact (hrs)** |
| **Annual review** | GP | 1 | 0.28 |
| **Titration visit and blood test** | Practice nurse | 1 | 0.25 |

**Appendix 3: Annual incremental expenditure on diabetes prevention programme as a % of current national expenditure on T2DM**

| **Years** | **IGT_pragmatic lifestyle** | **IGT_intensive lifestyle** | **IGT_metformin** | **IFG_pragmatic lifestyle** | **IFG_intensive lifestyle** | **IFG_metformin** | **HbA1c_pragmatic lifestyle** | **HbA1c_intensive lifestyle** | **HbA1c_metformin** |
| --- | --- | --- | --- | --- | --- | --- | --- | --- | --- |
| **1** | 0.5% | 3.1% | 0.3% | 0.9% | 5.2% | 0.5% | 0.7% | 4.4% | 0.4% |
| **2** | 0.2% | 1.4% | 0.3% | 0.3% | 2.3% | 0.4% | 0.2% | 1.9% | 0.4% |
| **3** | 0.0% | 1.0% | 0.2% | 0.0% | 1.8% | 0.4% | 0.0% | 1.5% | 0.3% |
| **4** | 0.0% | -0.1% | 0.2% | 0.0% | -0.1% | 0.3% | 0.0% | -0.1% | 0.2% |
| **5** | 0.0% | -0.1% | 0.1% | 0.0% | -0.1% | 0.2% | 0.0% | -0.1% | 0.2% |
| **6** | 0.0% | -0.1% | 0.1% | 0.0% | -0.1% | 0.2% | 0.0% | -0.1% | 0.1% |
| **7** | 0.0% | -0.2% | 0.1% | 0.0% | -0.2% | 0.1% | 0.0% | -0.1% | 0.1% |
| **8** | 0.0% | -0.2% | 0.0% | 0.0% | -0.2% | 0.1% | 0.0% | -0.1% | 0.0% |
| **9** | 0.0% | -0.2% | 0.0% | -0.1% | -0.2% | 0.1% | 0.0% | -0.2% | 0.0% |
| **10** | 0.0% | -0.2% | 0.0% | -0.1% | -0.2% | 0.0% | 0.0% | -0.2% | 0.0% |
| **11** | 0.0% | -0.2% | 0.0% | -0.1% | -0.2% | 0.0% | 0.0% | -0.2% | -0.1% |
| **12** | 0.0% | -0.2% | 0.0% | -0.1% | -0.2% | 0.0% | 0.0% | -0.2% | -0.1% |
| **13** | 0.0% | -0.2% | 0.0% | -0.1% | -0.3% | 0.0% | 0.0% | -0.2% | -0.1% |
| **14** | 0.0% | -0.2% | -0.1% | -0.1% | -0.3% | -0.1% | 0.0% | -0.2% | -0.1% |
| **15** | 0.0% | -0.2% | -0.1% | -0.1% | -0.3% | -0.1% | 0.0% | -0.2% | -0.1% |
| **16** | 0.0% | -0.2% | -0.1% | -0.1% | -0.3% | -0.1% | 0.0% | -0.2% | -0.1% |
| **17** | 0.0% | -0.2% | -0.1% | -0.1% | -0.3% | -0.1% | 0.0% | -0.2% | -0.1% |
| **18** | 0.0% | -0.2% | -0.1% | 0.0% | -0.2% | -0.1% | 0.0% | -0.2% | -0.1% |
| **19** | 0.0% | -0.2% | -0.1% | 0.0% | -0.2% | -0.1% | 0.0% | -0.2% | -0.1% |
| **20** | 0.0% | -0.2% | -0.1% | 0.0% | -0.2% | -0.1% | 0.0% | -0.2% | -0.1% |

**Appendix 4: Scenarios: Results of 1-way sensitivity analyses**

| **Method of identifying participants** | **Intervention** | **Base case** | | **Increase in duration of intervention effect*** | | **Decreased cost of intervention (80% of base case)** | | **Increased cost of intervention (120% of base case)** | | **WHO diagnostic criteria for IFG** | | **Unrelated healthcare costs included** | |
| --- | --- | --- | --- | --- | --- | --- | --- | --- | --- | --- | --- | --- | --- |
|  |  | **Cost (£)** | **QALYs** | **Cost (£)** | **QALYs** | **Cost (£)** | **QALYs** | **Cost (£)** | **QALYs** | **Cost (£)** | **QALYs** | **Cost (£)** | **QALYs** |
| **IGT** | **No intervention** | 17,772 | 11.53 | - | - | - | - | - | - | - | - | 17,772 | 11.53 |
|  | **Pragmatic lifestyle programme** | 17,774 | 11.59 | 17,587 | 11.60 | 17,717 | 11.59 | 17,832 | 11.59 | - | - | 17,826 | 11.59 |
|  | **Intensive lifestyle programme** | 18,423 | 11.76 | 18,329 | 11.78 | 17,984 | 11.76 | 18,862 | 11.76 | - | - | 18,857 | 11.76 |
|  | **Metformin** | 18,139 | 11.60 | 18,001 | 11.63 | 17,952 | 11.60 | 18,325 | 11.60 | - | - | 18,268 | 11.60 |
| **IFG** | **No intervention** | 17,429 | 12.13 | - | - | - | - | - | - | 18,426 | 12.04 | 17,429 | 12.13 |
|  | **Pragmatic lifestyle programme** | 17,440 | 12.19 | 17263 | 12.20 | 17,386 | 12.19 | 17,494 | 12.19 | 18,372 | 12.01 | 17,481 | 12.19 |
|  | **Intensive lifestyle programme** | 18,452 | 12.28 | 18,342 | 12.30 | 18,007 | 12.28 | 18,897 | 12.28 | 19,171 | 12.2 | 18,700 | 12.28 |
|  | **Metformin** | 17,908 | 12.20 | 17,747 | 12.24 | 17,703 | 12.20 | 18,041 | 12.20 | 18,690 | 12.12 | 18,016 | 12.20 |
| **HbA1c** | **No intervention** | 17,436 | 12.13 | - | - | - | - | - | - | - | - | 17,436 | 12.13 |
|  | **Pragmatic lifestyle programme** | 17,446 | 12.19 | 17,270 | 12.20 | 17,392 | 12.19 | 17,501 | 12.19 | - | - | 17,488 | 12.19 |
|  | **Intensive lifestyle programme** | 18,507 | 12.27 | 18,344 | 12.30 | 18,063 | 12.27 | 18,951 | 12.27 | - | - | 18,751 | 12.27 |
|  | **Metformin** | 17,475 | 12.23 | 17,227 | 12.28 | 17,266 | 12.23 | 17,684 | 12.23 | - | - | 17,663 | 12.23 |

*Intervention effect persists for 5 years with pragmatic lifestyle programme, and until the participant develops T2DM or reverts to normoglycaemia with intensive lifestyle programme or metformin

**Appendix 5: Probabilistic sensitivity analysis – scatter plots**

**4.1. Impaired glucose tolerance**


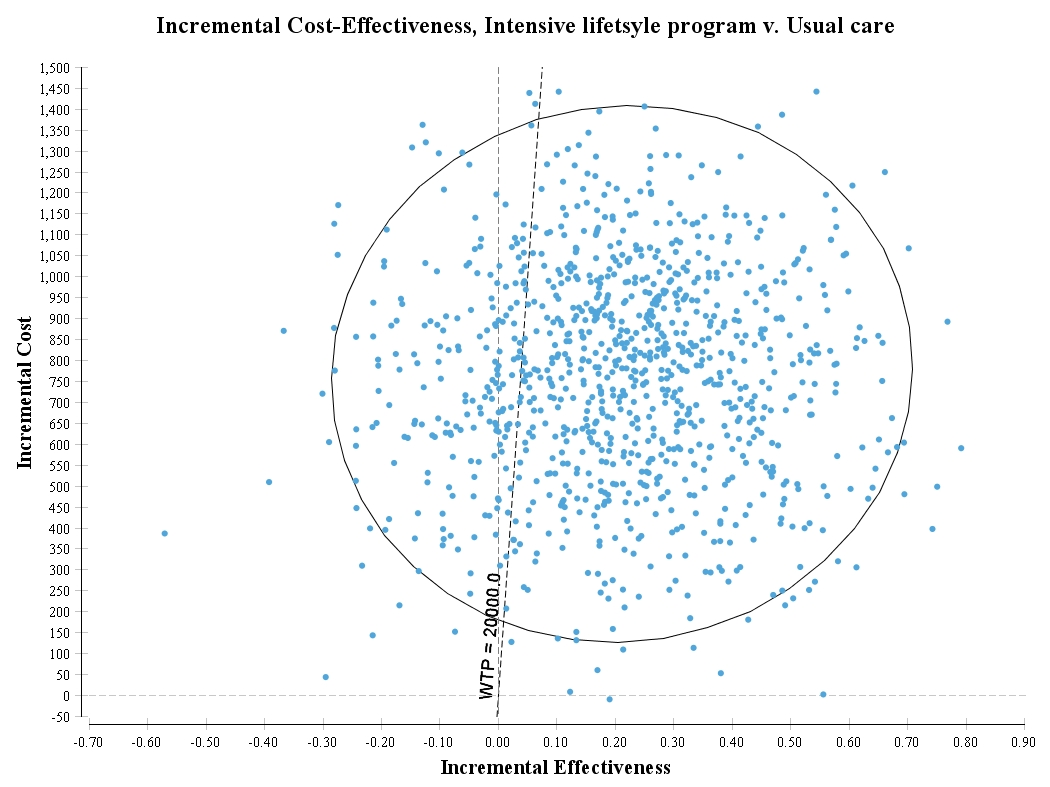

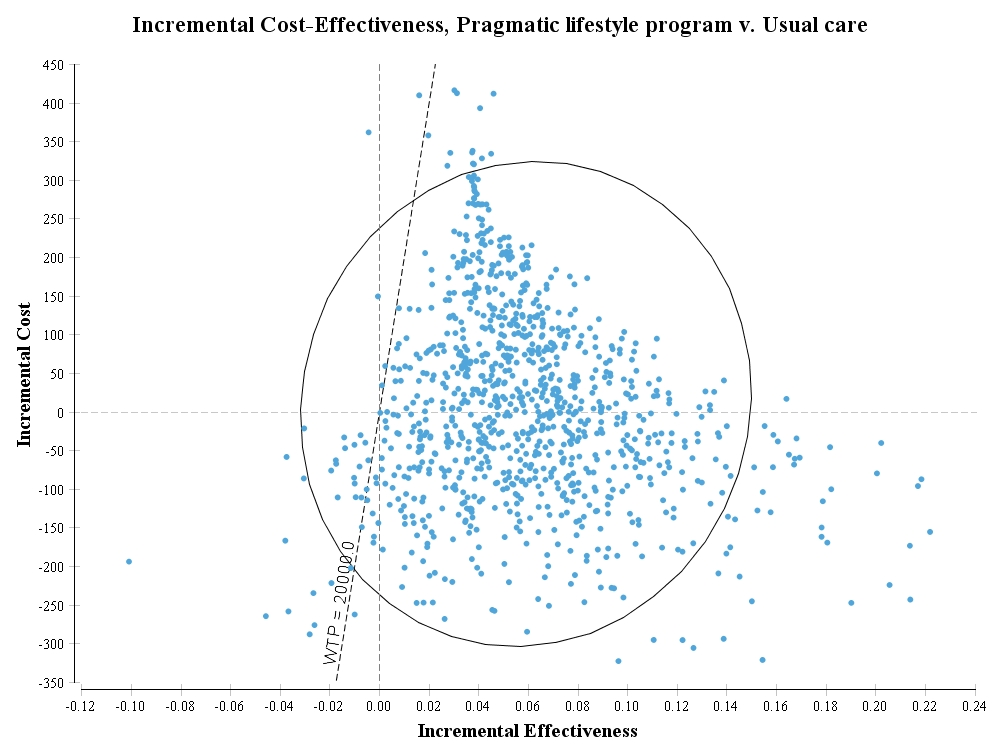


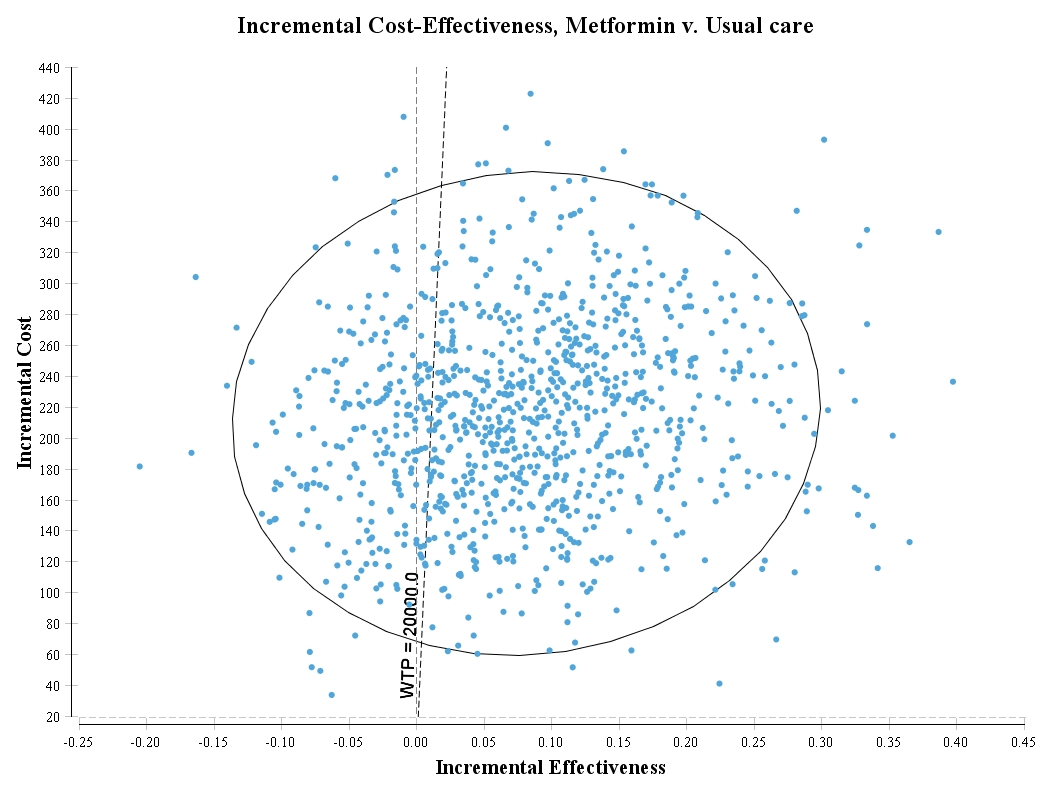


**4.2. Impaired fasting glucose**


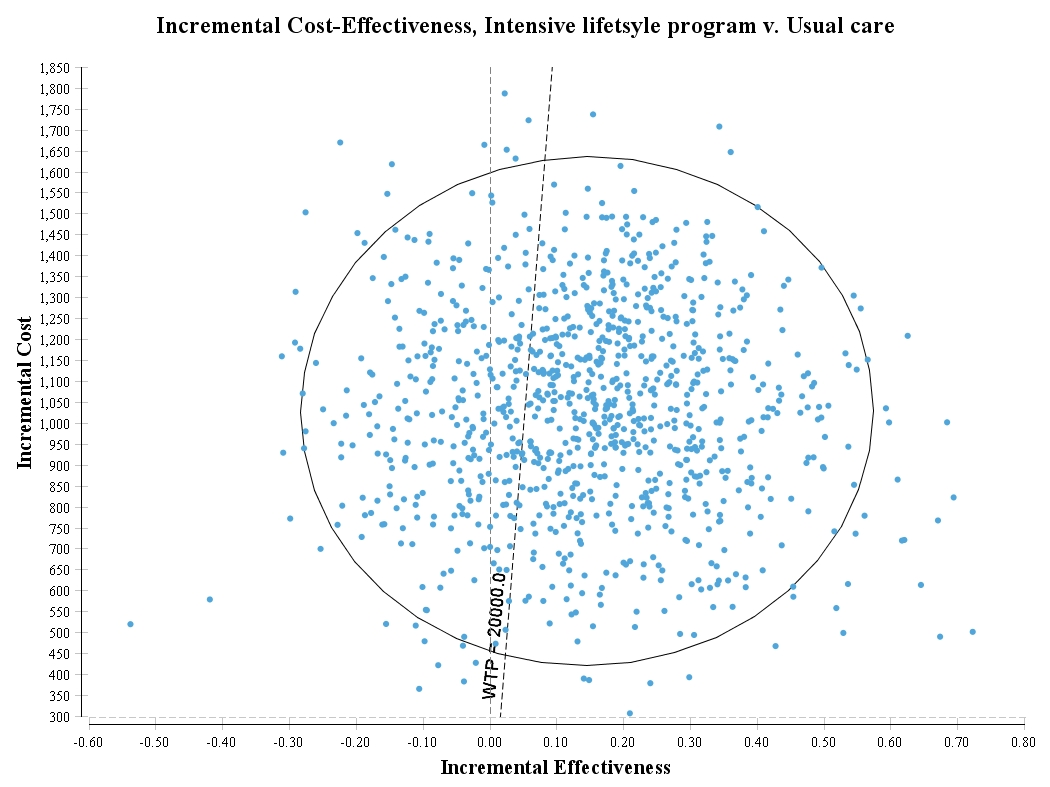

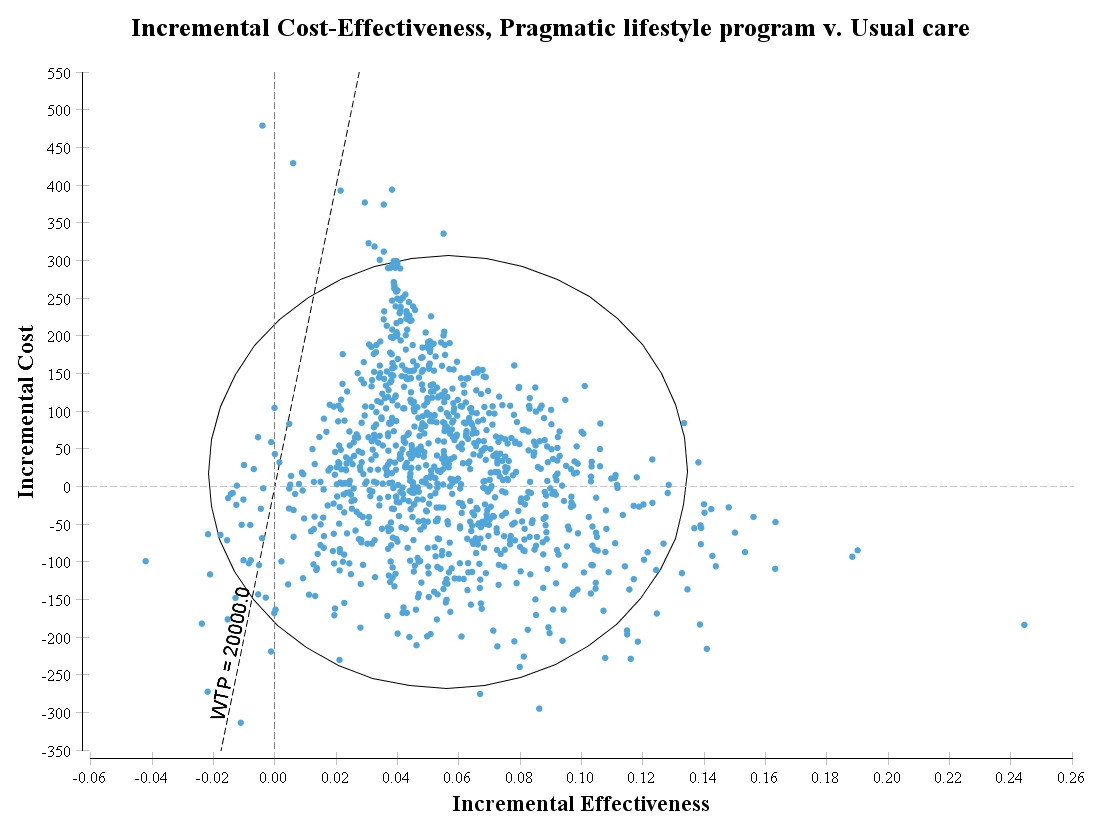


**Appendix 6: Assessment of the Validation Status of Health-Economic decision models (AdVISHE)**

**6.1. AdVISHE checklist**

| **PART A: VALIDATION OF THE CONCEPTUAL MODEL** |
| --- |
| **A1: Face validity testing of the conceptual model: Have experts been asked to judge the appropriateness of the conceptual model?** |
| Three experts were asked to judge face validity of the conceptual model, namely:   1. Prof Simon Griffin, Professor of General Practice, Institute of Public Health, University of Cambridge: Prof Griffin leads the Prevention of Diabetes and Related Metabolic Disorders Programme in the MRC Epidemiology unit at the University of Cambridge and has extensive experience in evaluating the impact of changes to diet and physical activity and their impact on obesity and diabetes. 2. Dr Amanda Adler, Chair of a Technology Appraisal Committee at the National Institute of Clinical Excellence (NICE) and consultant diabetologist at Addenbrooke’s Hospital, Cambridge: Amanda has both clinical knowledge of diabetes prevention and management and extensive experience in reviewing Markov models due to her role at NICE. 3. Dr Eleanor Barry, NIHR In-Practice Fellow, Department of Primary Care Health Science, University of Oxford and general practitioner: Eleanor has experience of implementing diabetes prevention programs through her work in general practice, was lead author of a recently published systematic review of efficacy of diabetes prevention programmes and is a Member of the Update Committee for the National Institute of Clinical Excellence’s guideline on diabetes prevention (PH38)   All agreed that the conceptual model accurately represented the underlying clinical process and was appropriate for economic evaluation. They recommended that the analysis be expanded to consider participants identified according to HbA1c as well as those identified on the basis of IFG and IGT. Prof Griffin noted that some patients with T2DM may revert to pre-diabetes for a period, but the large diabetes prevention trials do not report the incidence of pre-diabetes in those with T2DM, therefore we do not have the data to model this transition with a Markov model. |
| **A2. Cross validity testing (conceptual model): Has this model been compared to other conceptual models in the literature or clinical textbooks?** |
| The conceptual model was compared with 27 economic evaluations identified in our systematic review (Roberts, 2017). Of these evaluations, 20 were modelling studies and 14 of these models were Markov models. The conceptual model outlined in this paper is similar to these Markov models with two exceptions:   1. Earlier models did not include the transition from pre-diabetes to normal glucose tolerance, an area that has been addressed by more recent models and the model used for this study 2. Two different approaches were taken in the modelling of the T2DM health state across the different models: 1) model a health state of ‘uncomplicated’ T2DM and separate health states for complications of T2DM such as cardiovascular disease, renal disease and retinopathy, 2) model a single health state for T2DM and reflect that increasing incidence of diabetic complications by increasing annual costs of diabetes care. This study adopted the latter approach |
| **PART B: INPUT DATA VALIDATION** |
| **B1. Face validity testing (input data): Have experts been asked to judge the appropriateness of the input data?** |
| Experts outlined in A1 were asked to judge the appropriateness of the input data. They suggested a number of different data sources in the areas of increased mortality risk, prevalence of IFG and IGT, adherence to interventions and activities to be included in interventions. These additional data sources were incorporated in the final model. They noted the limitations in primary data associated with participants identified on the basis of HbA1c. In addition, they recommended a number of scenarios to be modeled in the sensitivity analysis where there is range of clinical practice (e.g. the type of metformin prescribed) or uncertainty due to lack of primary trial data (e.g. duration of relative risk reduction following pragmatic lifestyle programme). |
| **B2: Model fit testing: When input parameters are based on regression models, have statistical tests been performed?** |
| No input parameters were based on regression models |
| **PART C: VALIDATION OF THE COMPUTERISED MODEL** |
| **C1. External review: Has the computerized model been examined by modelling experts?** |
| The model was reviewed by a member of the TreeAge Support team to verify that the model structure was logical and that any inputs that required calculations or involved tunnel states were correctly coded. |
| **C2. Extreme value testing: Has the model been run for specific, extreme sets of parameter values in order to detect any coding errors?** |
| Extreme value testing was undertaken for extremely high and low values for the following parameters: mortality, costs of T2DM, relative risks of T2DM incidence in participants with pre-diabetes, costs of interventions. The results of extreme value testing of the IFG model are summarized in Appendix 5.2., with extreme values testing for the HbA1c and IGT model available from the authors. |
| **C3. Testing of traces: Have patients been tracked through the model to determine whether its logic is correct?** |
| Markov cohort traces were reviewed, with the example results for the IFG model included in Appendix 5.3. Cohort traces of the HbA1c and IGT model can be obtained from the authors. |
| **C4. Unit testing: Have individualized sub-modules of the computerized model been tested?** |
| Submodules of the model (e.g. normoglycaemic state) were turned on and off and global parameters such as discount rates or costs of T2DM were altered as outlined above. |
| **PART D: OPERATIONAL VALIDATION** |
| **D1. Face validity testing: Have experts been asked to judge the appropriateness of model outcomes?** |
| Experts outlined in A1 were asked to judge the appropriateness of model outcomes. They agreed that the model outcomes were appropriate and Dr Adler suggested the inclusion of ‘average number of years with T2DM’ as an additional outcome. This was incorporated into the model. |
| **D2. Cross validity testing: Have the model outcomes been compared to the outcomes of other models that address similar problems?** |
| Results of this model were compared with ICERs of existing economic evaluations obtained through a systematic review and converted to 2015 GBP using a PPP index (Roberts et al) and is reported in the discussion of this paper. |
| **D3. Validation against outcomes using alternative input data: Have the model outcomes been compared to the outcomes obtained using alternative input data?** |
| Yes, scenario analysis was undertaken and is reported in Appendix 3. |
| **D4: Validation against empirical data: Have the model outcomes been compared to empirical data?** |
| Yes, survival curves for all scenarios were plotted against national mortality data (example of IGT survival curve attached in Appendix 5.3 below) and the prevalence of T2DM we generated from our model was compared to National Diabetes Audit data for 2015-2016 (results in Appendix 5.4 below). |
| **E. OTHER VALIDATION TECHNIQUES** |
| **E1. Other validation techniques: Have any other validation techniques been performed?** |
| Yes, the model structure and parameter values were presented at two ‘work in progress’ meetings/seminars to health economists from other universities/departments. Their recommendations regarding model validation, sensitivity analyses and alternative sources for parameter values were incorporated in the final model. |

**Appendix Table 6.2. Example results of extreme value testing IFG model**

| **IFG Usual care** |  |  |  |  |  |  |  |
| --- | --- | --- | --- | --- | --- | --- | --- |
|  | **Base-case** | **pMortality: 0** | **pMortality: 0.1** | **RR_IFG_T2DM: 0.1** | **RR_IFG_T2DM: 1** | **cT2DM: £5,000** | **cT2DM: £100** |
| **Cost (£)** | £ 17,429 | £ 30,497 | £ 4,801 | £ 17,429 | £ 17,429 | £ 26,901 | £ 10,915 |
| **Efficacy (QALYs)** | 12.13 | 18.04 | 3.95 | 12.13 | 12.13 | 12.13 | 12.13 |
| **Years wth T2DM (number yrs)** | 5.34 | 17.57 | 0.61 | 5.34 | 5.34 | 5.34 | 5.34 |
| **Proportion with T2DM (%)** | 38% | 56% | 14% | 38% | 38% | 38% | 38% |
| **Proportion of 50 year olds who are dead at end of model (%)** | 100% | 0% | 100% | 100% | 100% | 100% | 100% |
|  |  |  |  |  |  |  |  |
| **IFG Pragmatic lifestyle** |  |  |  |  |  |  |  |
|  | **Base-case** | **pMortality: 0** | **pMortality: 0.1** | **RR_IFG_T2DM: 0.1** | **RR_IFG_T2DM: 1** | **cT2DM: £5,000** | **cT2DM: £100** |
| **Cost (£)** | £ 17,440 | £ 30,289 | £ 5,027 | £ 16,791 | £ 17,699 | £ 26,374 | £ 11,345 |
| **Efficacy (QALYs)** | 12.19 | 18.09 | 4.00 | 12.24 | 12.17 | 12.19 | 12.19 |
| **Years wth T2DM (number yrs)** | 5.07 | 17.02 | 0.55 | 0.00 | 5.34 | 5.07 | 5.07 |
| **Proportion with T2DM (%)** | 37% | 55% | 13% | 34% | 38% | 37% | 37% |
| **Proportion of 50 year olds who are dead at end of model (%)** | 100% | 0% | 100% | 100% | 100% | 100% | 100% |
|  |  |  |  |  |  |  |  |
| **IFG Intensive lifestyle** |  |  |  |  |  |  |  |
|  | **Base-case** | **pMortality: 0** | **pMortality: 0.1** | **RR_IFG_T2DM: 0.1** | **RR_IFG_T2DM: 1** | **cT2DM: £5,000** | **cT2DM: £100** |
| **Cost (£)** | £ 18,452 | £ 30,229 | £ 6,758 | £ 17,576 | £ 19,036 | £ 25,394 | £ 13,803 |
| **Efficacy (QALYs)** | 12.28 | 18.12 | 4.15 | 12.35 | 12.23 | 12.28 | 12.28 |
| **Years wth T2DM (number yrs)** | 3.98 | 14.47 | 0.42 | 2.98 | 4.64 | 3.98 | 3.98 |
| **Proportion with T2DM (%)** | 31% | 51% | 10% | 27% | 34% | 31% | 31% |
| **Proportion of 50 year olds who are dead at end of model (%)** | 100% | 0% | 100% | 100% | 100% | 100% | 100% |
|  |  |  |  |  |  |  |  |
| **IFG Metformin** |  |  |  |  |  |  |  |
|  | **Base-case** | **pMortality: 0** | **pMortality: 0.1** | **RR_IFG_T2DM: 0.1** | **RR_IFG_T2DM: 1** | **cT2DM: £5,000** | **cT2DM: £100** |
| **Cost (£)** | £ 17,775 | £ 30,367 | £ 5,192 | £ 15,325 | £ 18,409 | £ 25,759 | £ 12,374 |
| **Efficacy (QALYs)** | 12.21 | 18.09 | 3.99 | 12.50 | 12.15 | 12.21 | 12.21 |
| **Years wth T2DM (number yrs)** | 4.55 | 15.86 | 0.49 | 0.65 | 5.34 | 4.55 | 4.55 |
| **Proportion with T2DM (%)** | 34% | 53% | 11% | 5% | 38% | 34% | 34% |
| **Proportion of 50 year olds who are dead at end of model (%)** | 100% | 0% | 100% | 100% | 100% | 100% | 100% |
|  |  |  |  |  |  |  |  |
| **Comments** |  | Zero probability of death, increased cost, utility, years with T2DM and proportion of people with T2DM | 100% probability of death, lower costs, efficacy, proportion with T2DM and years with T2DM | Lower cost, years with T2Dm and proportion with T2DM and higher efficacy | Higher cost, years with T2DM and proportion with T2DM , lower efficacy | Increased cost but no change to other variables | Decreased cost but no change to other variables |
|  |  |  |  |  |  |  |  |
|  |  |  |  |  |  |  |  |
|  |  |  |  |  |  |  |  |
|  |  |  |  |  |  |  |  |
|  |  |  |  |  |  |  |  |
|  |  |  |  |  |  |  |  |
|  |  |  |  |  |  |  |  |
|  |  |  |  |  |  |  |  |

**Appendix Table 6.3. Cohort traces IFG model**

| **USUAL CARE** | | | | | | | | | | | | | | |
| --- | --- | --- | --- | --- | --- | --- | --- | --- | --- | --- | --- | --- | --- | --- |
| **Stage** | **% Normal Gluose tolerance** | **% Pre-diabetes** | **% Type 2 Diabetes Mellitus** | **% Dead** | **Stage Cost** | **Cumulative Cost** | **Stage Eff** | **Cumulative Eff** | **Stage Deaths** | **Cumulative Deaths** | **Stage Cases of T2DM** | **Cumulative Cases of T2DM** | **Stage Years with T2DM** | **Cumulative Years with T2DM** |
| 0 | 0 | 1 | 0 | 0 | 434.5 | 434.5 | 0.3795 | 0.3795 | 0.005266 | 0.005266 | 0.03554 | 0.03554 | 0 | 0 |
| 1 | 0.089687 | 0.869507 | 0.03554 | 0.005266 | 837.9565 | 1272.456 | 0.72953 | 1.10903 | 0.005938 | 0.011204 | 0.030902 | 0.066442 | 0.035234 | 0.035234 |
| 2 | 0.161037 | 0.761622 | 0.066137 | 0.011204 | 811.7354 | 2084.192 | 0.700652 | 1.809683 | 0.006605 | 0.01781 | 0.027068 | 0.09351 | 0.065505 | 0.100739 |
| 3 | 0.217337 | 0.672281 | 0.092573 | 0.01781 | 789.2729 | 2873.465 | 0.672393 | 2.482076 | 0.007265 | 0.025075 | 0.023893 | 0.117403 | 0.0916 | 0.192339 |
| 4 | 0.261297 | 0.598135 | 0.115493 | 0.025075 | 769.7109 | 3643.176 | 0.644775 | 3.126851 | 0.007916 | 0.032991 | 0.021258 | 0.138661 | 0.11417 | 0.306509 |
| 5 | 0.295146 | 0.536435 | 0.135428 | 0.032991 | 752.3575 | 4395.533 | 0.617817 | 3.744668 | 0.008556 | 0.041547 | 0.019065 | 0.157726 | 0.133748 | 0.440258 |
| 6 | 0.320722 | 0.484918 | 0.152813 | 0.041547 | 736.6565 | 5132.19 | 0.591532 | 4.336199 | 0.009422 | 0.050969 | 0.017234 | 0.17496 | 0.15072 | 0.590978 |
| 7 | 0.339464 | 0.441613 | 0.167954 | 0.050969 | 721.9694 | 5854.159 | 0.56579 | 4.901989 | 0.010269 | 0.061238 | 0.015695 | 0.190655 | 0.165436 | 0.756414 |
| 8 | 0.3526 | 0.405031 | 0.181131 | 0.061238 | 707.9438 | 6562.103 | 0.540618 | 5.442607 | 0.011094 | 0.072332 | 0.014395 | 0.205049 | 0.178182 | 0.934596 |
| 9 | 0.361144 | 0.373947 | 0.192577 | 0.072332 | 694.2995 | 7256.402 | 0.516038 | 5.958645 | 0.011893 | 0.084225 | 0.01329 | 0.21834 | 0.189193 | 1.123789 |
| 10 | 0.365934 | 0.347358 | 0.202483 | 0.084225 | 680.8161 | 7937.218 | 0.492071 | 6.450715 | 0.012665 | 0.09689 | 0.012345 | 0.230685 | 0.198664 | 1.322454 |
| 11 | 0.367662 | 0.324439 | 0.211009 | 0.09689 | 667.3235 | 8604.542 | 0.468735 | 6.91945 | 0.01419 | 0.11108 | 0.011531 | 0.242215 | 0.20651 | 1.528964 |
| 12 | 0.366625 | 0.304254 | 0.218041 | 0.11108 | 653.0637 | 9257.606 | 0.445652 | 7.365102 | 0.015649 | 0.126729 | 0.010813 | 0.253028 | 0.212857 | 1.741821 |
| 13 | 0.363295 | 0.286306 | 0.22367 | 0.126729 | 637.9689 | 9895.574 | 0.422892 | 7.787994 | 0.017034 | 0.143763 | 0.010175 | 0.263204 | 0.217804 | 1.959625 |
| 14 | 0.358072 | 0.270185 | 0.227979 | 0.143763 | 622.0061 | 10517.58 | 0.40052 | 8.188514 | 0.018336 | 0.1621 | 0.009602 | 0.272806 | 0.221443 | 2.181068 |
| 15 | 0.351296 | 0.255559 | 0.231046 | 0.1621 | 605.172 | 11122.75 | 0.378593 | 8.567107 | 0.019549 | 0.181649 | 0.009083 | 0.281889 | 0.223859 | 2.404926 |
| 16 | 0.343253 | 0.242157 | 0.232941 | 0.181649 | 585.5183 | 11708.27 | 0.357169 | 8.924276 | 0.021937 | 0.203585 | 0.008606 | 0.290495 | 0.224651 | 2.629577 |
| 17 | 0.333742 | 0.229415 | 0.233257 | 0.203585 | 562.7053 | 12270.98 | 0.335762 | 9.260038 | 0.02412 | 0.227706 | 0.008153 | 0.298648 | 0.223914 | 2.853491 |
| 18 | 0.323009 | 0.217218 | 0.232067 | 0.227706 | 537.4084 | 12808.38 | 0.314513 | 9.574552 | 0.02608 | 0.253785 | 0.00772 | 0.306368 | 0.22174 | 3.075231 |
| 19 | 0.311278 | 0.205477 | 0.22946 | 0.253785 | 510.2366 | 13318.62 | 0.293555 | 9.868107 | 0.027798 | 0.281583 | 0.007303 | 0.313671 | 0.218234 | 3.293465 |
| 20 | 0.29875 | 0.194129 | 0.225537 | 0.281583 | 481.7375 | 13800.36 | 0.273009 | 10.14112 | 0.029264 | 0.310847 | 0.006899 | 0.32057 | 0.21351 | 3.506975 |
| 21 | 0.285613 | 0.183131 | 0.220409 | 0.310847 | 452.4017 | 14252.76 | 0.252987 | 10.3941 | 0.032754 | 0.343601 | 0.006508 | 0.327079 | 0.206742 | 3.713717 |
| 22 | 0.271247 | 0.171901 | 0.21325 | 0.343601 | 421.0141 | 14673.77 | 0.232778 | 10.62688 | 0.035634 | 0.379236 | 0.006109 | 0.333188 | 0.198192 | 3.911909 |
| 23 | 0.255926 | 0.160537 | 0.204301 | 0.379236 | 388.3199 | 15062.09 | 0.212669 | 10.83955 | 0.037869 | 0.417105 | 0.005705 | 0.338894 | 0.188133 | 4.100041 |
| 24 | 0.239918 | 0.14914 | 0.193838 | 0.417105 | 355.024 | 15417.12 | 0.192923 | 11.03247 | 0.039445 | 0.45655 | 0.0053 | 0.344194 | 0.17686 | 4.276901 |
| 25 | 0.223481 | 0.137809 | 0.18216 | 0.45655 | 321.7735 | 15738.89 | 0.173772 | 11.20624 | 0.040369 | 0.496919 | 0.004898 | 0.349092 | 0.164681 | 4.441582 |
| 26 | 0.206858 | 0.126645 | 0.169578 | 0.496919 | 289.143 | 16028.03 | 0.155415 | 11.36166 | 0.043354 | 0.540273 | 0.004501 | 0.353593 | 0.150745 | 4.592327 |
| 27 | 0.189355 | 0.115126 | 0.155246 | 0.540273 | 255.9354 | 16283.97 | 0.137218 | 11.49888 | 0.044996 | 0.58527 | 0.004092 | 0.357684 | 0.135698 | 4.728025 |
| 28 | 0.171418 | 0.103522 | 0.13979 | 0.58527 | 223.1797 | 16507.15 | 0.119606 | 11.61848 | 0.045357 | 0.630627 | 0.003679 | 0.361364 | 0.120147 | 4.848171 |
| 29 | 0.153467 | 0.09208 | 0.123826 | 0.630627 | 191.7545 | 16698.9 | 0.102931 | 11.72141 | 0.044557 | 0.675184 | 0.003273 | 0.364636 | 0.104648 | 4.95282 |
| 30 | 0.13588 | 0.081015 | 0.107921 | 0.675184 | 162.3568 | 16861.26 | 0.087463 | 11.80888 | 0.042766 | 0.71795 | 0.002879 | 0.367515 | 0.089683 | 5.042503 |
| 31 | 0.118981 | 0.070507 | 0.092562 | 0.71795 | 135.4896 | 16996.75 | 0.073391 | 11.88227 | 0.043874 | 0.761824 | 0.002506 | 0.370021 | 0.074113 | 5.116616 |
| 32 | 0.101711 | 0.059846 | 0.076619 | 0.761824 | 109.5427 | 17106.29 | 0.059893 | 11.94216 | 0.042512 | 0.804337 | 0.002127 | 0.372148 | 0.059109 | 5.175725 |
| 33 | 0.084877 | 0.04955 | 0.061236 | 0.804337 | 85.84454 | 17192.14 | 0.047554 | 11.98972 | 0.039222 | 0.843558 | 0.001761 | 0.373909 | 0.045518 | 5.221243 |
| 34 | 0.069137 | 0.040025 | 0.047279 | 0.843558 | 65.24602 | 17257.38 | 0.036751 | 12.02647 | 0.034643 | 0.878202 | 0.001423 | 0.375332 | 0.033862 | 5.255105 |
| 35 | 0.054967 | 0.031547 | 0.035284 | 0.878202 | 48.1302 | 17305.51 | 0.027657 | 12.05412 | 0.029412 | 0.907613 | 0.001121 | 0.376453 | 0.024349 | 5.279453 |
| 36 | 0.042652 | 0.024265 | 0.02547 | 0.907613 | 34.48744 | 17340 | 0.02028 | 12.0744 | 0.022182 | 0.929796 | 0.000862 | 0.377315 | 0.017576 | 5.297029 |
| 37 | 0.033077 | 0.018689 | 0.018438 | 0.929796 | 24.77542 | 17364.78 | 0.014896 | 12.0893 | 0.016766 | 0.946562 | 0.000664 | 0.377979 | 0.012724 | 5.309753 |
| 38 | 0.02564 | 0.01441 | 0.013388 | 0.946562 | 17.84407 | 17382.62 | 0.01096 | 12.10026 | 0.012699 | 0.959261 | 0.000512 | 0.378492 | 0.009239 | 5.318991 |
| 39 | 0.019868 | 0.01112 | 0.009751 | 0.959261 | 12.88447 | 17395.51 | 0.008076 | 12.10834 | 0.009637 | 0.968898 | 0.000395 | 0.378887 | 0.006729 | 5.32572 |
| 40 | 0.015391 | 0.008587 | 0.007124 | 0.968898 | 9.326465 | 17404.83 | 0.00596 | 12.1143 | 0.007326 | 0.976224 | 0.000305 | 0.379192 | 0.004916 | 5.330636 |
| 41 | 0.011921 | 0.006634 | 0.005221 | 0.976224 | 6.767318 | 17411.6 | 0.004403 | 12.1187 | 0.005578 | 0.981802 | 0.000236 | 0.379428 | 0.003603 | 5.334239 |
| 42 | 0.009231 | 0.005128 | 0.003839 | 0.981802 | 4.92185 | 17416.52 | 0.003257 | 12.12196 | 0.004254 | 0.986057 | 0.000182 | 0.37961 | 0.002649 | 5.336888 |
| 43 | 0.007148 | 0.003964 | 0.002831 | 0.986057 | 3.587653 | 17420.11 | 0.002412 | 12.12437 | 0.003249 | 0.989306 | 0.000141 | 0.379751 | 0.001954 | 5.338842 |
| 44 | 0.005534 | 0.003066 | 0.002095 | 0.989306 | 2.620696 | 17422.73 | 0.001788 | 12.12616 | 0.002485 | 0.99179 | 0.000109 | 0.37986 | 0.001445 | 5.340288 |
| 45 | 0.004284 | 0.002371 | 0.001554 | 0.99179 | 1.918216 | 17424.65 | 0.001326 | 12.12748 | 0.001902 | 0.993692 | 8.43E-05 | 0.379944 | 0.001073 | 5.34136 |
| 46 | 0.003317 | 0.001834 | 0.001157 | 0.993692 | 1.406702 | 17426.05 | 0.000985 | 12.12847 | 0.001458 | 0.99515 | 6.52E-05 | 0.380009 | 0.000798 | 5.342159 |
| 47 | 0.002567 | 0.001419 | 0.000864 | 0.99515 | 1.033415 | 17427.09 | 0.000732 | 12.1292 | 0.001118 | 0.996268 | 5.04E-05 | 0.38006 | 0.000596 | 5.342754 |
| 48 | 0.001987 | 0.001098 | 0.000646 | 0.996268 | 0.760433 | 17427.85 | 0.000544 | 12.12974 | 0.000859 | 0.997127 | 3.9E-05 | 0.380099 | 0.000446 | 5.343201 |
| 49 | 0.001538 | 0.00085 | 0.000485 | 0.997127 | 0.560408 | 17428.41 | 0.000405 | 12.13015 | 0.00066 | 0.997787 | 3.02E-05 | 0.380129 | 0.000335 | 5.343535 |
| 50 | 0.001191 | 0.000657 | 0.000365 | 0.997787 | 0.206786 | 17428.62 | 0.000151 | 12.1303 | 0 | 0.997787 | 0 | 0.380129 | 0 | 5.343535 |
|  |  |  |  |  |  |  |  |  |  |  |  |  |  |  |
| **PRAGMATIC LIFESTYLE INTERVENTION** | | | | | | | | | | | | | | |
| **Stage** | **% Normal Gluose tolerance** | **% Pre-diabetes** | **% Type 2 Diabetes Mellitus** | **% Dead** | **Stage Cost** | **Cumulative Cost** | **Stage Eff** | **Cumulative Eff** | **Stage Deaths** | **Cumulative Deaths** | **Stage Cases of T2DM** | **Cumulative Cases of T2DM** | **Stage Years with T2DM** | **Cumulative Years with T2DM** |
| 0 | 0 | 1 | 0 | 0 | 637.94 | 637.94 | 0.38895 | 0.38895 | 0.005266 | 0.005266 | 0.0263 | 0.0263 | 0 | 0 |
| 1 | 0.089687 | 0.878747 | 0.0263 | 0.005266 | 903.1269 | 1541.067 | 0.745764 | 1.134714 | 0.005914 | 0.01118 | 0.023111 | 0.04941 | 0.026073 | 0.026073 |
| 2 | 0.161866 | 0.777771 | 0.049184 | 0.01118 | 805.7016 | 2346.769 | 0.714732 | 1.849446 | 0.006555 | 0.017734 | 0.027642 | 0.077052 | 0.048714 | 0.074787 |
| 3 | 0.219552 | 0.686357 | 0.076356 | 0.017734 | 781.6808 | 3128.449 | 0.67277 | 2.522216 | 0.007211 | 0.024945 | 0.024393 | 0.101445 | 0.075554 | 0.150342 |
| 4 | 0.264608 | 0.6105 | 0.099947 | 0.024945 | 760.7822 | 3889.232 | 0.645171 | 3.167388 | 0.007859 | 0.032805 | 0.021697 | 0.123143 | 0.098802 | 0.249144 |
| 5 | 0.299316 | 0.54738 | 0.1205 | 0.032805 | 742.2913 | 4631.523 | 0.618231 | 3.785619 | 0.008497 | 0.041301 | 0.019454 | 0.142596 | 0.119005 | 0.368149 |
| 6 | 0.325555 | 0.494685 | 0.138459 | 0.041301 | 725.6321 | 5357.155 | 0.591964 | 4.377583 | 0.009359 | 0.05066 | 0.017581 | 0.160178 | 0.136562 | 0.504711 |
| 7 | 0.3448 | 0.450396 | 0.154144 | 0.05066 | 710.1538 | 6067.309 | 0.56624 | 4.943822 | 0.010203 | 0.060863 | 0.016007 | 0.176185 | 0.151833 | 0.656544 |
| 8 | 0.358307 | 0.41299 | 0.16784 | 0.060863 | 695.4889 | 6762.798 | 0.541084 | 5.484907 | 0.011025 | 0.071888 | 0.014678 | 0.190862 | 0.165107 | 0.821651 |
| 9 | 0.367116 | 0.381211 | 0.179785 | 0.071888 | 681.3437 | 7444.142 | 0.516521 | 6.001428 | 0.011822 | 0.083711 | 0.013548 | 0.204411 | 0.176626 | 0.998277 |
| 10 | 0.372082 | 0.354034 | 0.190174 | 0.083711 | 667.4854 | 8111.627 | 0.49257 | 6.493998 | 0.012592 | 0.096303 | 0.012582 | 0.216993 | 0.186587 | 1.184864 |
| 11 | 0.373914 | 0.330614 | 0.19917 | 0.096303 | 653.732 | 8765.359 | 0.469249 | 6.963247 | 0.014112 | 0.110415 | 0.01175 | 0.228743 | 0.194923 | 1.379787 |
| 12 | 0.372918 | 0.309994 | 0.206673 | 0.110415 | 639.3342 | 9404.693 | 0.446182 | 7.409428 | 0.015567 | 0.125982 | 0.011017 | 0.23976 | 0.201759 | 1.581546 |
| 13 | 0.369578 | 0.291664 | 0.212776 | 0.125982 | 624.2138 | 10028.91 | 0.423437 | 7.832866 | 0.016949 | 0.142931 | 0.010366 | 0.250126 | 0.207196 | 1.788742 |
| 14 | 0.364301 | 0.275206 | 0.217562 | 0.142931 | 608.3271 | 10637.23 | 0.40108 | 8.233945 | 0.018249 | 0.16118 | 0.009781 | 0.259907 | 0.211324 | 2.000067 |
| 15 | 0.357435 | 0.260279 | 0.221105 | 0.16118 | 591.6608 | 11228.9 | 0.379168 | 8.613113 | 0.019461 | 0.180642 | 0.00925 | 0.269157 | 0.214228 | 2.214294 |
| 16 | 0.349275 | 0.246606 | 0.223478 | 0.180642 | 572.7688 | 11801.66 | 0.357756 | 8.970869 | 0.021844 | 0.202486 | 0.008764 | 0.277921 | 0.215524 | 2.429818 |
| 17 | 0.339615 | 0.233611 | 0.224288 | 0.202486 | 551.1561 | 12352.82 | 0.336361 | 9.30723 | 0.024025 | 0.226511 | 0.008303 | 0.286224 | 0.215304 | 2.645123 |
| 18 | 0.328708 | 0.221174 | 0.223607 | 0.226511 | 526.9927 | 12879.81 | 0.315125 | 9.622355 | 0.025985 | 0.252496 | 0.007861 | 0.294084 | 0.213656 | 2.858779 |
| 19 | 0.31678 | 0.209207 | 0.221517 | 0.252496 | 500.8872 | 13380.7 | 0.294176 | 9.916531 | 0.027706 | 0.280202 | 0.007435 | 0.30152 | 0.21068 | 3.069459 |
| 20 | 0.30404 | 0.197643 | 0.218115 | 0.280202 | 473.387 | 13854.09 | 0.273638 | 10.19017 | 0.029176 | 0.309378 | 0.007024 | 0.308544 | 0.206484 | 3.275943 |
| 21 | 0.290677 | 0.186437 | 0.213508 | 0.309378 | 444.9822 | 14299.07 | 0.253621 | 10.44379 | 0.032667 | 0.342045 | 0.006626 | 0.31517 | 0.200268 | 3.476211 |
| 22 | 0.276062 | 0.174999 | 0.206894 | 0.342045 | 414.4929 | 14713.56 | 0.233415 | 10.67721 | 0.035553 | 0.377598 | 0.006219 | 0.321389 | 0.192285 | 3.668496 |
| 23 | 0.260473 | 0.163425 | 0.198504 | 0.377598 | 382.6519 | 15096.21 | 0.213307 | 10.89051 | 0.037798 | 0.415396 | 0.005808 | 0.327197 | 0.182794 | 3.85129 |
| 24 | 0.244183 | 0.151818 | 0.188602 | 0.415396 | 350.1545 | 15446.37 | 0.193556 | 11.08407 | 0.039388 | 0.454783 | 0.005396 | 0.332593 | 0.172083 | 4.023373 |
| 25 | 0.227456 | 0.140281 | 0.177479 | 0.454783 | 317.6402 | 15764.01 | 0.174396 | 11.25846 | 0.040328 | 0.495112 | 0.004986 | 0.337579 | 0.160448 | 4.183822 |
| 26 | 0.21054 | 0.128915 | 0.165434 | 0.495112 | 285.6787 | 16049.69 | 0.156025 | 11.41449 | 0.043332 | 0.538444 | 0.004582 | 0.34216 | 0.14706 | 4.330882 |
| 27 | 0.192727 | 0.117188 | 0.151642 | 0.538444 | 253.0968 | 16302.78 | 0.137808 | 11.5523 | 0.044997 | 0.583441 | 0.004165 | 0.346325 | 0.132548 | 4.46343 |
| 28 | 0.174472 | 0.105375 | 0.136713 | 0.583441 | 220.9089 | 16523.69 | 0.120171 | 11.67247 | 0.045383 | 0.628824 | 0.003745 | 0.35007 | 0.117503 | 4.580933 |
| 29 | 0.156202 | 0.093727 | 0.121248 | 0.628824 | 189.9839 | 16713.68 | 0.103464 | 11.77593 | 0.044611 | 0.673435 | 0.003331 | 0.353401 | 0.102469 | 4.683402 |
| 30 | 0.138301 | 0.082463 | 0.1058 | 0.673435 | 161.0146 | 16874.69 | 0.08796 | 11.86389 | 0.042845 | 0.71628 | 0.002931 | 0.356332 | 0.08792 | 4.771322 |
| 31 | 0.121102 | 0.071767 | 0.090851 | 0.71628 | 134.5038 | 17009.2 | 0.073846 | 11.93774 | 0.043987 | 0.760267 | 0.002551 | 0.358883 | 0.072743 | 4.844065 |
| 32 | 0.103524 | 0.060915 | 0.075294 | 0.760267 | 108.8646 | 17118.06 | 0.060301 | 11.99804 | 0.042656 | 0.802923 | 0.002165 | 0.361047 | 0.058087 | 4.902152 |
| 33 | 0.08639 | 0.050435 | 0.060252 | 0.802923 | 85.414 | 17203.47 | 0.04791 | 12.04595 | 0.039388 | 0.842311 | 0.001792 | 0.36284 | 0.044787 | 4.946939 |
| 34 | 0.07037 | 0.04074 | 0.046579 | 0.842311 | 65.00098 | 17268.47 | 0.037053 | 12.083 | 0.034823 | 0.877134 | 0.001448 | 0.364288 | 0.03336 | 4.980299 |
| 35 | 0.055947 | 0.032111 | 0.034808 | 0.877134 | 48.01385 | 17316.49 | 0.027906 | 12.11091 | 0.029594 | 0.906728 | 0.001141 | 0.365429 | 0.02402 | 5.004319 |
| 36 | 0.043412 | 0.024698 | 0.025161 | 0.906728 | 34.45265 | 17350.94 | 0.020478 | 12.13139 | 0.022342 | 0.92907 | 0.000878 | 0.366307 | 0.017363 | 5.021682 |
| 37 | 0.033667 | 0.019023 | 0.018241 | 0.92907 | 24.78402 | 17375.73 | 0.015053 | 12.14644 | 0.016902 | 0.945972 | 0.000676 | 0.366983 | 0.012587 | 5.03427 |
| 38 | 0.026097 | 0.014667 | 0.013264 | 0.945972 | 17.87345 | 17393.6 | 0.011083 | 12.15752 | 0.012813 | 0.958785 | 0.000521 | 0.367504 | 0.009153 | 5.043422 |
| 39 | 0.020222 | 0.011319 | 0.009674 | 0.958785 | 12.92167 | 17406.52 | 0.008172 | 12.1657 | 0.009731 | 0.968516 | 0.000402 | 0.367906 | 0.006676 | 5.050098 |
| 40 | 0.015666 | 0.00874 | 0.007078 | 0.968516 | 9.364401 | 17415.88 | 0.006034 | 12.17173 | 0.007403 | 0.975919 | 0.000311 | 0.368217 | 0.004884 | 5.054983 |
| 41 | 0.012133 | 0.006753 | 0.005195 | 0.975919 | 6.802407 | 17422.69 | 0.00446 | 12.17619 | 0.005641 | 0.98156 | 0.00024 | 0.368457 | 0.003585 | 5.058568 |
| 42 | 0.009396 | 0.005219 | 0.003825 | 0.98156 | 4.952557 | 17427.64 | 0.003301 | 12.17949 | 0.004305 | 0.985865 | 0.000185 | 0.368642 | 0.002639 | 5.061207 |
| 43 | 0.007275 | 0.004035 | 0.002825 | 0.985865 | 3.613587 | 17431.25 | 0.002445 | 12.18194 | 0.003289 | 0.989154 | 0.000143 | 0.368786 | 0.001949 | 5.063156 |
| 44 | 0.005633 | 0.00312 | 0.002093 | 0.989154 | 2.642066 | 17433.9 | 0.001813 | 12.18375 | 0.002517 | 0.991671 | 0.000111 | 0.368897 | 0.001444 | 5.064601 |
| 45 | 0.004361 | 0.002414 | 0.001555 | 0.991671 | 1.935513 | 17435.83 | 0.001346 | 12.18509 | 0.001928 | 0.993598 | 8.58E-05 | 0.368983 | 0.001073 | 5.065674 |
| 46 | 0.003376 | 0.001867 | 0.001159 | 0.993598 | 1.420513 | 17437.25 | 0.001 | 12.18609 | 0.001478 | 0.995076 | 6.64E-05 | 0.369049 | 0.0008 | 5.066474 |
| 47 | 0.002613 | 0.001444 | 0.000866 | 0.995076 | 1.044328 | 17438.3 | 0.000743 | 12.18684 | 0.001134 | 0.996211 | 5.13E-05 | 0.3691 | 0.000598 | 5.067071 |
| 48 | 0.002023 | 0.001118 | 0.000649 | 0.996211 | 0.768982 | 17439.06 | 0.000553 | 12.18739 | 0.000871 | 0.997082 | 3.97E-05 | 0.36914 | 0.000448 | 5.067519 |
| 49 | 0.001566 | 0.000865 | 0.000488 | 0.997082 | 0.567061 | 17439.63 | 0.000411 | 12.1878 | 0.00067 | 0.997752 | 3.07E-05 | 0.369171 | 0.000336 | 5.067855 |
| 50 | 0.001212 | 0.000669 | 0.000367 | 0.997752 | 0.20936 | 17439.84 | 0.000153 | 12.18795 | 0 | 0.997752 | 0 | 0.369171 | 0 | 5.067855 |
|  |  |  |  |  |  |  |  |  |  |  |  |  |  |  |
| **INTENSIVE LIFESTYLE INTERVENTION** | | | | | | | | | | | | | | |
| **Stage** | **% Normal Gluose tolerance** | **% Pre-diabetes** | **% Type 2 Diabetes Mellitus** | **% Dead** | **Stage Cost** | **Cumulative Cost** | **Stage Eff** | **Cumulative Eff** | **Stage Deaths** | **Cumulative Deaths** | **Stage Cases of T2DM** | **Cumulative Cases of T2DM** | **Stage Years with T2DM** | **Cumulative Years with T2DM** |
| 0 | 0 | 1 | 0 | 0 | 1659.5 | 1659.5 | 0.38895 | 0.38895 | 0.005266 | 0.005266 | 0.02239 | 0.02239 | 0 | 0 |
| 1 | 0.137222 | 0.835122 | 0.02239 | 0.005266 | 1385.778 | 3045.278 | 0.745461 | 1.134411 | 0.005878 | 0.011144 | 0.018699 | 0.041089 | 0.022198 | 0.022198 |
| 2 | 0.241668 | 0.706292 | 0.040896 | 0.011144 | 1238.508 | 4283.786 | 0.714329 | 1.84874 | 0.006483 | 0.017626 | 0.015814 | 0.056903 | 0.040505 | 0.062703 |
| 3 | 0.320567 | 0.605488 | 0.056319 | 0.017626 | 766.5033 | 5050.289 | 0.674044 | 2.522783 | 0.00708 | 0.024706 | 0.013557 | 0.07046 | 0.055728 | 0.118431 |
| 4 | 0.379558 | 0.526451 | 0.069285 | 0.024706 | 740.0816 | 5790.371 | 0.646792 | 3.169575 | 0.00767 | 0.032376 | 0.014968 | 0.085428 | 0.068491 | 0.186922 |
| 5 | 0.423044 | 0.461121 | 0.083459 | 0.032376 | 716.921 | 6507.292 | 0.620097 | 3.789673 | 0.008261 | 0.040638 | 0.013111 | 0.098538 | 0.082424 | 0.269346 |
| 6 | 0.454019 | 0.409809 | 0.095535 | 0.040638 | 695.7872 | 7203.079 | 0.594047 | 4.38372 | 0.009073 | 0.04971 | 0.011652 | 0.11019 | 0.094226 | 0.363572 |
| 7 | 0.455738 | 0.388674 | 0.105878 | 0.04971 | 677.4131 | 7880.492 | 0.568388 | 4.952108 | 0.009884 | 0.059595 | 0.011051 | 0.121241 | 0.104291 | 0.467863 |
| 8 | 0.455059 | 0.370005 | 0.115341 | 0.059595 | 659.708 | 8540.2 | 0.543314 | 5.495422 | 0.010675 | 0.07027 | 0.01052 | 0.131761 | 0.113463 | 0.581327 |
| 9 | 0.452389 | 0.353358 | 0.123983 | 0.07027 | 642.5224 | 9182.723 | 0.518845 | 6.014267 | 0.011444 | 0.081714 | 0.010047 | 0.141807 | 0.121805 | 0.703131 |
| 10 | 0.448069 | 0.338365 | 0.131852 | 0.081714 | 625.7325 | 9808.455 | 0.494997 | 6.509265 | 0.012187 | 0.093902 | 0.012026 | 0.153833 | 0.129365 | 0.832496 |
| 11 | 0.442385 | 0.322323 | 0.14139 | 0.093902 | 609.7479 | 10418.2 | 0.47175 | 6.981015 | 0.013674 | 0.107576 | 0.011455 | 0.165288 | 0.138376 | 0.970872 |
| 12 | 0.435034 | 0.307559 | 0.149831 | 0.107576 | 593.5539 | 11011.76 | 0.448768 | 7.429783 | 0.015103 | 0.122679 | 0.010931 | 0.176219 | 0.146269 | 1.11714 |
| 13 | 0.426288 | 0.293834 | 0.157199 | 0.122679 | 577.0789 | 11588.84 | 0.426113 | 7.855896 | 0.016465 | 0.139143 | 0.010443 | 0.186662 | 0.153077 | 1.270217 |
| 14 | 0.416382 | 0.280955 | 0.163519 | 0.139143 | 560.2745 | 12149.11 | 0.403846 | 8.259742 | 0.017752 | 0.156895 | 0.009985 | 0.196647 | 0.158831 | 1.429048 |
| 15 | 0.405522 | 0.268766 | 0.168817 | 0.156895 | 543.1125 | 12692.22 | 0.382022 | 8.641765 | 0.018958 | 0.175853 | 0.009552 | 0.206199 | 0.163565 | 1.592614 |
| 16 | 0.393884 | 0.257146 | 0.173117 | 0.175853 | 524.3416 | 13216.56 | 0.360693 | 9.002458 | 0.02131 | 0.197163 | 0.009139 | 0.215338 | 0.166956 | 1.75957 |
| 17 | 0.381111 | 0.245631 | 0.176095 | 0.197163 | 503.5445 | 13720.11 | 0.339384 | 9.341842 | 0.023475 | 0.220638 | 0.00873 | 0.224068 | 0.169041 | 1.928611 |
| 18 | 0.367389 | 0.234202 | 0.177771 | 0.220638 | 481.1656 | 14201.27 | 0.31823 | 9.660072 | 0.025431 | 0.24607 | 0.008324 | 0.232391 | 0.16986 | 2.098471 |
| 19 | 0.352892 | 0.222855 | 0.178184 | 0.24607 | 457.5958 | 14658.87 | 0.297356 | 9.957428 | 0.027163 | 0.273233 | 0.00792 | 0.240311 | 0.169467 | 2.267938 |
| 20 | 0.337785 | 0.211596 | 0.177387 | 0.273233 | 433.0476 | 15091.92 | 0.276879 | 10.23431 | 0.028656 | 0.301889 | 0.00752 | 0.247832 | 0.167928 | 2.435865 |
| 21 | 0.322222 | 0.200441 | 0.175448 | 0.301889 | 407.8833 | 15499.8 | 0.256907 | 10.49121 | 0.032147 | 0.334036 | 0.007124 | 0.254955 | 0.164568 | 2.600433 |
| 22 | 0.305462 | 0.18881 | 0.171692 | 0.334036 | 380.9864 | 15880.79 | 0.236738 | 10.72795 | 0.035061 | 0.369097 | 0.00671 | 0.261666 | 0.159568 | 2.760001 |
| 23 | 0.28778 | 0.176845 | 0.166278 | 0.369097 | 352.9111 | 16233.7 | 0.21665 | 10.9446 | 0.037358 | 0.406455 | 0.006285 | 0.267951 | 0.153119 | 2.91312 |
| 24 | 0.26945 | 0.164691 | 0.159404 | 0.406455 | 324.2007 | 16557.9 | 0.196896 | 11.1415 | 0.039023 | 0.445478 | 0.005853 | 0.273804 | 0.145442 | 3.058562 |
| 25 | 0.250738 | 0.152489 | 0.151295 | 0.445478 | 295.3712 | 16853.27 | 0.177705 | 11.3192 | 0.040056 | 0.485535 | 0.005419 | 0.279223 | 0.136777 | 3.195339 |
| 26 | 0.231897 | 0.140372 | 0.142196 | 0.485535 | 266.8394 | 17120.11 | 0.159275 | 11.47848 | 0.043157 | 0.528692 | 0.004989 | 0.284212 | 0.126404 | 3.321743 |
| 27 | 0.212131 | 0.127784 | 0.131393 | 0.528692 | 237.5343 | 17357.64 | 0.140969 | 11.61945 | 0.044949 | 0.573641 | 0.004541 | 0.288753 | 0.114848 | 3.436591 |
| 28 | 0.191931 | 0.115039 | 0.11939 | 0.573641 | 208.3685 | 17566.01 | 0.123208 | 11.74266 | 0.045481 | 0.619122 | 0.004088 | 0.292842 | 0.102614 | 3.539205 |
| 29 | 0.171753 | 0.102422 | 0.106702 | 0.619122 | 180.1412 | 17746.15 | 0.106344 | 11.849 | 0.044861 | 0.663983 | 0.00364 | 0.296482 | 0.090177 | 3.629381 |
| 30 | 0.152014 | 0.090187 | 0.093817 | 0.663983 | 153.506 | 17899.66 | 0.09065 | 11.93965 | 0.043241 | 0.707224 | 0.003205 | 0.299687 | 0.077962 | 3.707343 |
| 31 | 0.133068 | 0.078541 | 0.081167 | 0.707224 | 128.954 | 18028.61 | 0.076319 | 12.01597 | 0.044569 | 0.751793 | 0.002791 | 0.302479 | 0.064989 | 3.772333 |
| 32 | 0.113726 | 0.066701 | 0.067781 | 0.751793 | 105.0205 | 18133.63 | 0.062523 | 12.07849 | 0.043411 | 0.795204 | 0.002371 | 0.304849 | 0.052291 | 3.824624 |
| 33 | 0.094884 | 0.055251 | 0.054661 | 0.795204 | 82.95222 | 18216.59 | 0.049855 | 12.12835 | 0.040279 | 0.835483 | 0.001964 | 0.306813 | 0.040631 | 3.865255 |
| 34 | 0.077275 | 0.044647 | 0.042595 | 0.835483 | 63.5819 | 18280.17 | 0.038707 | 12.16705 | 0.035794 | 0.871277 | 0.001587 | 0.308399 | 0.030506 | 3.895761 |
| 35 | 0.061429 | 0.035201 | 0.032093 | 0.871277 | 47.32315 | 18327.49 | 0.029272 | 12.19633 | 0.030582 | 0.901859 | 0.001251 | 0.309651 | 0.022147 | 3.917908 |
| 36 | 0.047661 | 0.027082 | 0.023398 | 0.901859 | 34.22743 | 18361.72 | 0.021571 | 12.2179 | 0.023211 | 0.92507 | 0.000962 | 0.310613 | 0.016146 | 3.934054 |
| 37 | 0.036959 | 0.020863 | 0.017109 | 0.92507 | 24.80914 | 18386.53 | 0.015918 | 12.23382 | 0.017647 | 0.942717 | 0.000741 | 0.311354 | 0.011806 | 3.94586 |
| 38 | 0.028647 | 0.016088 | 0.012548 | 0.942717 | 18.02087 | 18404.55 | 0.011762 | 12.24558 | 0.013439 | 0.956156 | 0.000572 | 0.311926 | 0.008659 | 3.954519 |
| 39 | 0.022197 | 0.012417 | 0.009231 | 0.956156 | 13.11743 | 18417.67 | 0.008701 | 12.25428 | 0.010249 | 0.966405 | 0.000441 | 0.312368 | 0.00637 | 3.960888 |
| 40 | 0.017195 | 0.009589 | 0.006811 | 0.966405 | 9.567658 | 18427.23 | 0.006443 | 12.26072 | 0.007828 | 0.974233 | 0.000341 | 0.312708 | 0.0047 | 3.965588 |
| 41 | 0.013317 | 0.007409 | 0.005041 | 0.974233 | 6.992238 | 18434.23 | 0.004776 | 12.2655 | 0.005986 | 0.980219 | 0.000263 | 0.312972 | 0.003479 | 3.969067 |
| 42 | 0.010313 | 0.005727 | 0.003742 | 0.980219 | 5.119693 | 18439.35 | 0.003543 | 12.26904 | 0.004583 | 0.984802 | 0.000204 | 0.313175 | 0.002582 | 3.971649 |
| 43 | 0.007985 | 0.004428 | 0.002786 | 0.984802 | 3.755337 | 18443.1 | 0.002631 | 12.27167 | 0.003512 | 0.988314 | 0.000157 | 0.313333 | 0.001922 | 3.973571 |
| 44 | 0.006182 | 0.003424 | 0.00208 | 0.988314 | 2.759233 | 18445.86 | 0.001955 | 12.27363 | 0.002695 | 0.991009 | 0.000122 | 0.313454 | 0.001435 | 3.975007 |
| 45 | 0.004786 | 0.002649 | 0.001557 | 0.991009 | 2.03057 | 18447.89 | 0.001453 | 12.27508 | 0.002069 | 0.993078 | 9.41E-05 | 0.313548 | 0.001074 | 3.976081 |
| 46 | 0.003705 | 0.002049 | 0.001168 | 0.993078 | 1.496556 | 18449.39 | 0.001081 | 12.27616 | 0.00159 | 0.994668 | 7.28E-05 | 0.313621 | 0.000806 | 3.976887 |
| 47 | 0.002868 | 0.001585 | 0.000879 | 0.994668 | 1.104498 | 18450.49 | 0.000805 | 12.27697 | 0.001223 | 0.995891 | 5.63E-05 | 0.313678 | 0.000607 | 3.977494 |
| 48 | 0.00222 | 0.001227 | 0.000663 | 0.995891 | 0.816182 | 18451.31 | 0.000599 | 12.27757 | 0.000941 | 0.996832 | 4.36E-05 | 0.313721 | 0.000458 | 3.977951 |
| 49 | 0.001718 | 0.000949 | 0.000501 | 0.996832 | 0.603827 | 18451.91 | 0.000447 | 12.27801 | 0.000725 | 0.997556 | 3.37E-05 | 0.313755 | 0.000346 | 3.978297 |
| 50 | 0.00133 | 0.000734 | 0.00038 | 0.997556 | 0.223597 | 18452.14 | 0.000166 | 12.27818 | 0 | 0.997556 | 0 | 0.313755 | 0 | 3.978297 |
|  |  |  |  |  |  |  |  |  |  |  |  |  |  |  |
| **METFORMIN** | | | | | | | | | | | | | | |
| Stage | % Normal Gluose tolerance | % Pre-diabetes | % Type 2 Diabetes Mellitus | % Dead | Stage Cost | Cumulative Cost | Stage Eff | Cumulative Eff | Stage Deaths | Cumulative Deaths | Stage Cases of T2DM | Cumulative Cases of T2DM | Stage Years with T2DM | Cumulative Years with T2DM |
| 0 | 0 | 1 | 0 | 0 | 558.75 | 558.75 | 0.38105 | 0.38105 | 0.005266 | 0.005266 | 0.0263 | 0.0263 | 0 | 0 |
| 1 | 0.089687 | 0.878747 | 0.0263 | 0.005266 | 940.6796 | 1499.43 | 0.73235 | 1.1134 | 0.005914 | 0.01118 | 0.023111 | 0.04941 | 0.026073 | 0.026073 |
| 2 | 0.161866 | 0.777771 | 0.049184 | 0.01118 | 895.9142 | 2395.344 | 0.70326 | 1.81666 | 0.006555 | 0.017734 | 0.020455 | 0.069865 | 0.048714 | 0.074787 |
| 3 | 0.219552 | 0.693544 | 0.069169 | 0.017734 | 857.3934 | 3252.737 | 0.674845 | 2.491505 | 0.007188 | 0.024922 | 0.01824 | 0.088105 | 0.068443 | 0.14323 |
| 4 | 0.265252 | 0.623143 | 0.086683 | 0.024922 | 823.8565 | 4076.594 | 0.647118 | 3.138623 | 0.007812 | 0.032734 | 0.016388 | 0.104494 | 0.08569 | 0.22892 |
| 5 | 0.301045 | 0.564143 | 0.102078 | 0.032734 | 794.2858 | 4870.879 | 0.620088 | 3.758711 | 0.008425 | 0.041158 | 0.014837 | 0.11933 | 0.100812 | 0.329732 |
| 6 | 0.328656 | 0.514537 | 0.115649 | 0.041158 | 767.8609 | 5638.74 | 0.593762 | 4.352473 | 0.009261 | 0.050419 | 0.013532 | 0.132863 | 0.114065 | 0.443797 |
| 7 | 0.349442 | 0.472542 | 0.127597 | 0.050419 | 743.7277 | 6382.468 | 0.568006 | 4.920479 | 0.010077 | 0.060496 | 0.012428 | 0.14529 | 0.125684 | 0.569481 |
| 8 | 0.364574 | 0.436818 | 0.138112 | 0.060496 | 721.3671 | 7103.835 | 0.542841 | 5.46332 | 0.010872 | 0.071369 | 0.011488 | 0.156778 | 0.135863 | 0.705344 |
| 9 | 0.375025 | 0.406255 | 0.147351 | 0.071369 | 700.3626 | 7804.198 | 0.518287 | 5.981607 | 0.011643 | 0.083012 | 0.014438 | 0.171217 | 0.144762 | 0.850106 |
| 10 | 0.381608 | 0.37618 | 0.1592 | 0.083012 | 680.8763 | 8485.074 | 0.494295 | 6.475902 | 0.012409 | 0.095421 | 0.013369 | 0.184586 | 0.156198 | 1.006303 |
| 11 | 0.38466 | 0.350352 | 0.169567 | 0.095421 | 662.4115 | 9147.485 | 0.470943 | 6.946845 | 0.013915 | 0.109335 | 0.012452 | 0.197038 | 0.165952 | 1.172255 |
| 12 | 0.384554 | 0.327707 | 0.178403 | 0.109335 | 644.0837 | 9791.569 | 0.447858 | 7.394703 | 0.015359 | 0.124695 | 0.011647 | 0.208684 | 0.174161 | 1.346417 |
| 13 | 0.381831 | 0.307667 | 0.185808 | 0.124695 | 625.7043 | 10417.27 | 0.425105 | 7.819808 | 0.016733 | 0.141428 | 0.010934 | 0.219619 | 0.180935 | 1.527352 |
| 14 | 0.376947 | 0.289755 | 0.19187 | 0.141428 | 607.1369 | 11024.41 | 0.402743 | 8.222551 | 0.018029 | 0.159457 | 0.010298 | 0.229917 | 0.186369 | 1.713721 |
| 15 | 0.370291 | 0.273586 | 0.196667 | 0.159457 | 588.2892 | 11612.7 | 0.38083 | 8.603381 | 0.019239 | 0.178695 | 0.009723 | 0.23964 | 0.190549 | 1.90427 |
| 16 | 0.36219 | 0.258842 | 0.200273 | 0.178695 | 567.6478 | 12180.35 | 0.359419 | 8.9628 | 0.021609 | 0.200304 | 0.009199 | 0.248839 | 0.193145 | 2.097415 |
| 17 | 0.352453 | 0.244899 | 0.202344 | 0.200304 | 544.6923 | 12725.04 | 0.338028 | 9.300828 | 0.023783 | 0.224087 | 0.008704 | 0.257543 | 0.194239 | 2.291654 |
| 18 | 0.341354 | 0.231617 | 0.202943 | 0.224087 | 519.8958 | 13244.94 | 0.316797 | 9.617625 | 0.025742 | 0.249829 | 0.008232 | 0.265775 | 0.193912 | 2.485565 |
| 19 | 0.329141 | 0.218887 | 0.202143 | 0.249829 | 493.6972 | 13738.63 | 0.295852 | 9.913477 | 0.027468 | 0.277296 | 0.007779 | 0.273554 | 0.192254 | 2.677819 |
| 20 | 0.31604 | 0.206631 | 0.200033 | 0.277296 | 466.5026 | 14205.14 | 0.275314 | 10.18879 | 0.028948 | 0.306244 | 0.007344 | 0.280897 | 0.189366 | 2.867185 |
| 21 | 0.302256 | 0.19479 | 0.19671 | 0.306244 | 438.6852 | 14643.82 | 0.255291 | 10.44408 | 0.032439 | 0.338684 | 0.006923 | 0.28782 | 0.184512 | 3.051697 |
| 22 | 0.287141 | 0.18274 | 0.191435 | 0.338684 | 409.021 | 15052.84 | 0.235077 | 10.67916 | 0.035337 | 0.374021 | 0.006495 | 0.294315 | 0.177917 | 3.229614 |
| 23 | 0.270991 | 0.170577 | 0.184411 | 0.374021 | 378.146 | 15430.99 | 0.214954 | 10.89411 | 0.037605 | 0.411626 | 0.006062 | 0.300377 | 0.169817 | 3.399431 |
| 24 | 0.254094 | 0.158402 | 0.175879 | 0.411626 | 346.6734 | 15777.66 | 0.19518 | 11.08929 | 0.039227 | 0.450853 | 0.00563 | 0.306007 | 0.160474 | 3.559905 |
| 25 | 0.236726 | 0.146318 | 0.166104 | 0.450853 | 315.0762 | 16092.74 | 0.175986 | 11.26528 | 0.040208 | 0.49106 | 0.0052 | 0.311207 | 0.150165 | 3.71007 |
| 26 | 0.219148 | 0.134427 | 0.155365 | 0.49106 | 283.9179 | 16376.65 | 0.157571 | 11.42285 | 0.043253 | 0.534313 | 0.004778 | 0.315984 | 0.13811 | 3.848179 |
| 27 | 0.200628 | 0.122171 | 0.142887 | 0.534313 | 252.0567 | 16628.71 | 0.139297 | 11.56215 | 0.044972 | 0.579286 | 0.004342 | 0.320326 | 0.124896 | 3.973075 |
| 28 | 0.181641 | 0.109836 | 0.129238 | 0.579286 | 220.4862 | 16849.2 | 0.121589 | 11.68374 | 0.045421 | 0.624707 | 0.003904 | 0.32423 | 0.111078 | 4.084153 |
| 29 | 0.162632 | 0.09768 | 0.114981 | 0.624707 | 190.0641 | 17039.26 | 0.104798 | 11.78853 | 0.044714 | 0.669421 | 0.003472 | 0.327701 | 0.097173 | 4.181326 |
| 30 | 0.144003 | 0.08593 | 0.100645 | 0.669421 | 161.4804 | 17200.74 | 0.089197 | 11.87773 | 0.043011 | 0.712432 | 0.003054 | 0.330755 | 0.083636 | 4.264963 |
| 31 | 0.1261 | 0.074777 | 0.08669 | 0.712432 | 135.2431 | 17335.99 | 0.074977 | 11.95271 | 0.044233 | 0.756665 | 0.002658 | 0.333413 | 0.069412 | 4.334374 |
| 32 | 0.107802 | 0.063464 | 0.072069 | 0.756665 | 109.7874 | 17445.77 | 0.061311 | 12.01402 | 0.042976 | 0.79964 | 0.002256 | 0.335669 | 0.055599 | 4.389974 |
| 33 | 0.089963 | 0.052542 | 0.057855 | 0.79964 | 86.42374 | 17532.2 | 0.04879 | 12.06281 | 0.039766 | 0.839407 | 0.001867 | 0.337536 | 0.043005 | 4.432978 |
| 34 | 0.073282 | 0.042439 | 0.044872 | 0.839407 | 66.00954 | 17598.21 | 0.037797 | 12.10061 | 0.035236 | 0.874642 | 0.001508 | 0.339044 | 0.032138 | 4.465116 |
| 35 | 0.058263 | 0.033448 | 0.033646 | 0.874642 | 48.95208 | 17647.16 | 0.028518 | 12.12912 | 0.030014 | 0.904657 | 0.001189 | 0.340233 | 0.023218 | 4.488334 |
| 36 | 0.045211 | 0.025726 | 0.024407 | 0.904657 | 35.27502 | 17682.43 | 0.020966 | 12.15009 | 0.022711 | 0.927368 | 0.000914 | 0.341147 | 0.016842 | 4.505177 |
| 37 | 0.035061 | 0.019814 | 0.017757 | 0.927368 | 25.47945 | 17707.91 | 0.015439 | 12.16553 | 0.017219 | 0.944587 | 0.000704 | 0.341851 | 0.012253 | 4.51743 |
| 38 | 0.027178 | 0.015277 | 0.012958 | 0.944587 | 18.44713 | 17726.36 | 0.011385 | 12.17691 | 0.013079 | 0.957666 | 0.000543 | 0.342394 | 0.008942 | 4.526372 |
| 39 | 0.02106 | 0.011789 | 0.009485 | 0.957666 | 13.3864 | 17739.75 | 0.008407 | 12.18532 | 0.009952 | 0.967618 | 0.000419 | 0.342813 | 0.006545 | 4.532917 |
| 40 | 0.016315 | 0.009103 | 0.006964 | 0.967618 | 9.735741 | 17749.48 | 0.006215 | 12.19154 | 0.007584 | 0.975201 | 0.000324 | 0.343137 | 0.004806 | 4.537722 |
| 41 | 0.012636 | 0.007033 | 0.005129 | 0.975201 | 7.095968 | 17756.58 | 0.0046 | 12.19614 | 0.005788 | 0.980989 | 0.00025 | 0.343387 | 0.00354 | 4.541262 |
| 42 | 0.009786 | 0.005436 | 0.003789 | 0.980989 | 5.182664 | 17761.76 | 0.003408 | 12.19954 | 0.004423 | 0.985412 | 0.000193 | 0.34358 | 0.002615 | 4.543877 |
| 43 | 0.007577 | 0.004203 | 0.002808 | 0.985412 | 3.792717 | 17765.55 | 0.002527 | 12.20207 | 0.003384 | 0.988797 | 0.000149 | 0.343729 | 0.001938 | 4.545815 |
| 44 | 0.005866 | 0.00325 | 0.002087 | 0.988797 | 2.780725 | 17768.33 | 0.001876 | 12.20395 | 0.002592 | 0.991389 | 0.000116 | 0.343845 | 0.00144 | 4.547255 |
| 45 | 0.004542 | 0.002514 | 0.001556 | 0.991389 | 2.042341 | 17770.38 | 0.001393 | 12.20534 | 0.001988 | 0.993377 | 8.93E-05 | 0.343934 | 0.001074 | 4.548329 |
| 46 | 0.003516 | 0.001945 | 0.001163 | 0.993377 | 1.502493 | 17771.88 | 0.001036 | 12.20638 | 0.001526 | 0.994903 | 6.91E-05 | 0.344003 | 0.000803 | 4.549131 |
| 47 | 0.002721 | 0.001504 | 0.000872 | 0.994903 | 1.107029 | 17772.99 | 0.00077 | 12.20715 | 0.001172 | 0.996074 | 5.35E-05 | 0.344057 | 0.000601 | 4.549733 |
| 48 | 0.002107 | 0.001164 | 0.000655 | 0.996074 | 0.816803 | 17773.8 | 0.000573 | 12.20772 | 0.000901 | 0.996975 | 4.14E-05 | 0.344098 | 0.000452 | 4.550185 |
| 49 | 0.001631 | 0.000901 | 0.000493 | 0.996975 | 0.603443 | 17774.41 | 0.000427 | 12.20815 | 0.000693 | 0.997668 | 3.2E-05 | 0.34413 | 0.00034 | 4.550525 |
| 50 | 0.001262 | 0.000697 | 0.000372 | 0.997668 | 0.215418 | 17774.62 | 0.000159 | 12.2083 | 0 | 0.997668 | 0 | 0.34413 | 0 | 4.550525 |

**Appendix 6.4 Survival curve analysis – IGT example**

**Appendix 6.5. Comparison of T2DM prevalence generated from the model and National Diabetes Audit prevalence data**

|  | **Prevalence of T2DM by age** | | | | | | |
| --- | --- | --- | --- | --- | --- | --- | --- |
|  | **55-59 years** | **60-64 years** | **65-69 years** | **70-74 years** | **75-79 years** | **80-84 years** | **85+ years** |
| **Model output*** | 6.8% | 10.5% | 11.6% | 10.9% | 8.8% | 5.8% | 3.2% |
| **National Diabetes Audit 2015-2016 [42]+ prevalence of undiagnosed T2DM [43]**** | 7.5% | 8.4% | 9.8% | 8.7% | 7.9% | 6.0% | 4.7% |

*****Incident cases of T2DM derived from IFG, IGT and HbA1c combined according to percentage prevalence of different types of intermediate hyperglycaemia

** National Diabetes Audit raw data only collects data on people registered as diabetic with their GP. Public Health England estimates 25% of people with T2DM are not diagnosed [43]. This proportion of undiagnosed diabetes has been added to the National Diabetes Audit data in the table above
